# Developing a survey tool to assess gendered violence and mental health burden in migrants living in precarity

**DOI:** 10.1136/bmjopen-2026-120912

**Published:** 2026-09-30

**Authors:** Neha Adsul, Roomi Aziz, Muthusamy Sivakami, Kamaldeep Bhui, Anuj Kapilashrami, GEMMS Global Health Research Group, Kapilashrami Anuj

**Affiliations:** 1 Tata Institute of Social Sciences, Mumbai, Maharashtra, India; 2 Centre for Global Health and Intersectional Equity Research, University of Essex, Colchester, UK; 3 University of Oxford, Oxford, UK; 4 University of Essex, Colchester, UK

**Keywords:** MENTAL HEALTH, Refugees, Gender-Based Violence, Surveys and Questionnaires, Community Participation

## Abstract

**Abstract:**

**Background:**

Migrants living in precarious conditions often face gendered violence (GV) and poor mental health (MH). However, most of the existing GV and MH measures assume residentially stable populations, missing migration-related precarity or structural conditions shaping migrants’ experiences. As a result, the intersection of GV, poor MH and precarity across migrant populations remains unstudied, especially in low- and middle-income country (LMIC) settings, that the National Institute for Health and Care Research (NIHR)-funded research programme Gendered Violence and Poor Mental Health among Migrants in Precarious Situations (GEMMS) aims to explore.

**Aim:**

This paper describes the development of the GEMMS survey tool and presents preliminary pilot validation evidence for selected adapted and newly developed components designed to measure GV, MH and precarity associated with migration across multiple LMIC settings.

**Methods:**

GEMMS survey tool development followed a participatory process with multicountry research teams, international experts, local advisory groups and community members with lived experiences. Guided by GEMMS’ conceptual frameworks, items were drawn from validated instruments where possible and newly developed as informed by GEMMS’ ethnography and country profiling. After several rounds of expert reviews, community consultations and item-level content validation, the tool was pilot-tested in Cambodia, India and South Africa (total n=198), to improve wording, sequencing, skip logics and order. Exploratory factor analysis was done to assess internal coherence of the newly adapted items.

**Results:**

Final survey tool comprises 216 items. Exploratory factor analysis of selected adapted and de novo migration-related and psychosocial item sets in pilot analyses identified two coherent migration-related and three MH-related factors: structural harm, socio-cultural marginalisation; social exclusion, belonging/social integration and migration stress. All five factors were robust, with interpretable and meaningful loadings that aligned with the guiding theory.

**Conclusion:**

The GEMMS survey tool provides an integrated, context-responsive, structurally informed survey tool for measuring GV, MH and multidimensional precarity in migrant populations. This paper demonstrates its development process, pilot feasibility and preliminary validation evidence for selected components, while establishing a foundation for future intersectional and syndemic-informed analysis across diverse migrant populations in LMICs.

**STRENGTHS AND LIMITATIONS OF THIS STUDY**

- The survey tool development involved an iterative, participatory process that engaged international experts and advisors, country researchers, community-based local advisory groups and migrants, specifically for the low- and middle-income country context.
- The tool has been designed to capture structural and migration-related drivers that may shape the co-occurrence of poor mental health and gendered violence, setting a foundation for future syndemic and intersectional analysis
- While designed as a uniform multicountry tool, not all items are directly comparable at the item level; some country-specific categories were introduced to capture country-level intersectional granularities relevant to the country’s context.
- In addition to documenting the process, this study provides early validation evidence for selected adapted and de novo items; complete psychometric validation, measurement invariance testing of the full survey data and formal analysis of syndemic and intersectional relationships is still needed and requires full survey sample.

## Background

 Migrants are often exposed to multiple and overlapping forms of vulnerability across their migration trajectories, including precarious employment, insecure legal status, discrimination, poor living conditions and restricted access to services. These experiences can contribute to a reinforcing cycle of poor mental health (MH), gendered violence (GV) and socioeconomic disadvantage. Migration-related precarity, including status-related, work-related and governance-related forms of insecurity, shapes both exposure to harm and the resources available to cope with it, creating layered experiences of vulnerability and exclusion.^1^

Evidence increasingly demonstrates that poor MH and experiences of violence frequently co-occur among migrant populations, particularly among those living in precarious circumstances. These experiences are not produced in isolation but are shaped by broader structural, social and political conditions. This is where ‘Syndemic theory’ offers a useful lens for understanding how multiple adverse conditions interact and reinforce one another within contexts of inequality, while intersectionality highlights how factors such as gender, migration status, class, ethnicity and other social locations shape differential exposure to these harms.^2 3^

However, existing measurement approaches remain fragmented in the literature. Quantitative research on migrant health has expanded in recent years, with increasing number of studies estimating the prevalence of violence exposure or specific MH conditions. Although existing violence surveys often prioritise individual or incident level reporting, universalising violence as a personal problem, erases structural and contextual complexities.^4 5^ Similarly, many widely used MH tools have several methodological constraints and developmental limitations, such as being rooted in Western psychological frameworks and inaccurately estimating MH prevalence due to cultural inappropriateness.^6 7^ Generally, most of these instruments are designed in high-income countries settings, and assume residentially stable populations, disregarding diverse migration contexts, including literacy, immigration status and gender and lacking the nuance to fully represent migration experiences of psychological distress,^8^ missing low- and middle-income country (LMIC) contexts.

Most existing survey tools fail to account for mobile populations and tend to overlook migration-specific experiences, such as the broader social, structural and contextual conditions like labour circumstances, mobility cycles, indebtedness, income precarity and risk factors, which are crucial in shaping access and help-seeking behaviours in contexts like precarious labour, documentation insecurity, illegal travel, etc.^9–11^ Specifically, due to siloed measurement, overlooking of structural drivers and not accommodating heterogeneity within migrants’ context, their utility for informing policy and programme design for migrants remains constrained in LMICs’ context.

There are also several limitations within the current evidence base. While multipurpose largescale household surveys have incorporated social determinants and related risk factors and drivers of poor MH^12 13^; they rarely offer a way to examine co-occurrence or interaction across the spectrum of GV and poor MH. As a result, the co-occurrence and mutually reinforcing nature of these conditions remain obscured within conventional survey approaches. This is despite the growing evidence of co-occurrence and interconnectedness of GV and poor MH, and the centrality of this evidence for responsive intervention design for migrant populations, especially in the LMICs (Tennyson, Kemp and Rao, 2016; Mapitsa, 2020). Existing tools typically focus on individual domains, specific migrant groups or single-country contexts, limiting their ability to examine how structural vulnerability, violence and mental health interact across different migration trajectories and sociopolitical environments.^14–16^

To address this methodological gap, the NIHR-funded Gendered Violence and Poor Mental Health among Migrants in Precarious Situations (GEMMS) project developed a multicountry modular survey tool informed by syndemic and intersectionality perspectives. The survey was designed to capture migration-related precarity, GV, MH and related structural determinants within a single measurement framework across India, Cambodia, South Africa and Zimbabwe. This paper describes the conceptual foundations, participatory development process, content validation, pilot testing and preliminary validation findings for selected components of the GEMMS survey tool.

### Aims

This paper documents the conceptual foundations, methodological steps, participatory review approaches and adaptation principles used in developing the GEMMS survey tool, and presents preliminary validation findings for selected adapted and newly developed components. The resulting approach offers flexibility, allowing other researchers to evolve and refine this contextually and structurally informed modular survey tool. The specific aims were:

- To develop a syndemic-informed and intersectionality-informed contextually adaptable multicountry survey tool capable of capturing the relationships between migration-related precarity, GV, MH and psychosocial distress across diverse migrant populations in LMIC settings.
- To generate preliminary evidence regarding the feasibility, content validity and internal coherence of selected adapted and newly developed survey components prior to implementation in the full GEMMS study.

## Methodology

This section describes the conceptual framework, survey development process, item generation strategy, content validation, pilot testing and preliminary validation procedures used to develop the GEMMS survey tool.

This study has been reported in accordance with CROSS checklist (Consensus-based Checklist for Reporting of Survey Studies) for survey studies, with relevant COSMIN (COnsensus-based Standards for the selection of health Measurement INstruments) reporting guidance applied to the measurement property components, particularly for content validity, structural validity and internal consistency (see online supplemental checklist).

### Patient and public involvement

As detailed later, in addition to experts and researchers, community advisory groups and migrants with lived experiences were involved in consultation, feedback, item identification, local contextualisation and validation of items, as well as post-pilot discussion and tool review.

### Conceptual framework guiding the survey tool development

The GEMMS survey tool was developed using syndemicity, intersectionality and migration-related precarity as its guiding conceptual frameworks.^1^ These frameworks shaped both the overall structure of the tool and the selection of domains and subdomains within it.

Migration-related precarity was conceptualised as extending beyond economic deprivation to accommodate status-based, labour-related, social and governance-related insecurities that shape migrants’ lived experiences and exposure to harm. Operationalising this framework required not only addressing GV and MH outcomes, but also addressing the broader social, economic, legal and political conditions through which vulnerability is produced and sustained.

Syndemicity informed the decision to measure GV, MH and migration-related precarity within a single instrument, recognising that these conditions frequently co-occur and interact through structural inequalities. Accordingly, the survey tool was designed to capture not only experiences of GV and MH symptoms, but also the conditions under which these harms may cluster. These included employment insecurity, housing conditions, documentation status, access to state and humanitarian assistance, service exclusion, mobility histories, discrimination, harassment and broader indicators of structural vulnerability. In this way, the survey tool enabled moving beyond estimating single-domain prevalence and examining the relationships among migration-related precarity, GV and psychosocial distress.

Intersectionality informed the inclusion of variables needed to examine how vulnerability differs across intersecting social positions and migration trajectories^17 18^ and the decision to retain selected country-specific categories where necessary, all within this single survey tool. This shaped the inclusion of demographic and social identity variables, including gender, age, education, social group or ethnicity (where contextually relevant), relationship status, migration history, documentation status, labour conditions, housing circumstances and access to services.

Together, these frameworks guided the development of a modular survey tool that combines standardised measurement with contextual adaptation across India, Cambodia, South Africa and Zimbabwe.

### Overall survey development process and a summary of the timeline

The process of survey tool development began in March 2024, after completion of the formative phase of GEMMS, which included detailed country site situation assessments, rapid ethnography and transect walks, systematic reviews, country profiling, policy profiling and policy indexing.^19 20^ Following the conceptual guidance of the GEMMS proposal,^21^ and informed by the formative work, the initial drafts of the survey tool were developed by the GEMMS India team (NA and MS), with critical revisions by the GEMMS UK team (RA, AK and KB) and international expert advisory group members contributing to choice of tools and the rationale. Partnering country teams contributed to instrument selection, feasibility and using culturally appropriate language and terminology across study settings. The survey tool was finalised by RA, NA, MS and AK, and then piloted across three country sites in November 2024. Based on the piloting experience and teams’ feedback, the tool was refined and locked by RA and NA in December 2024 for multicountry roll-out (see figure 1).

**Figure 1:**
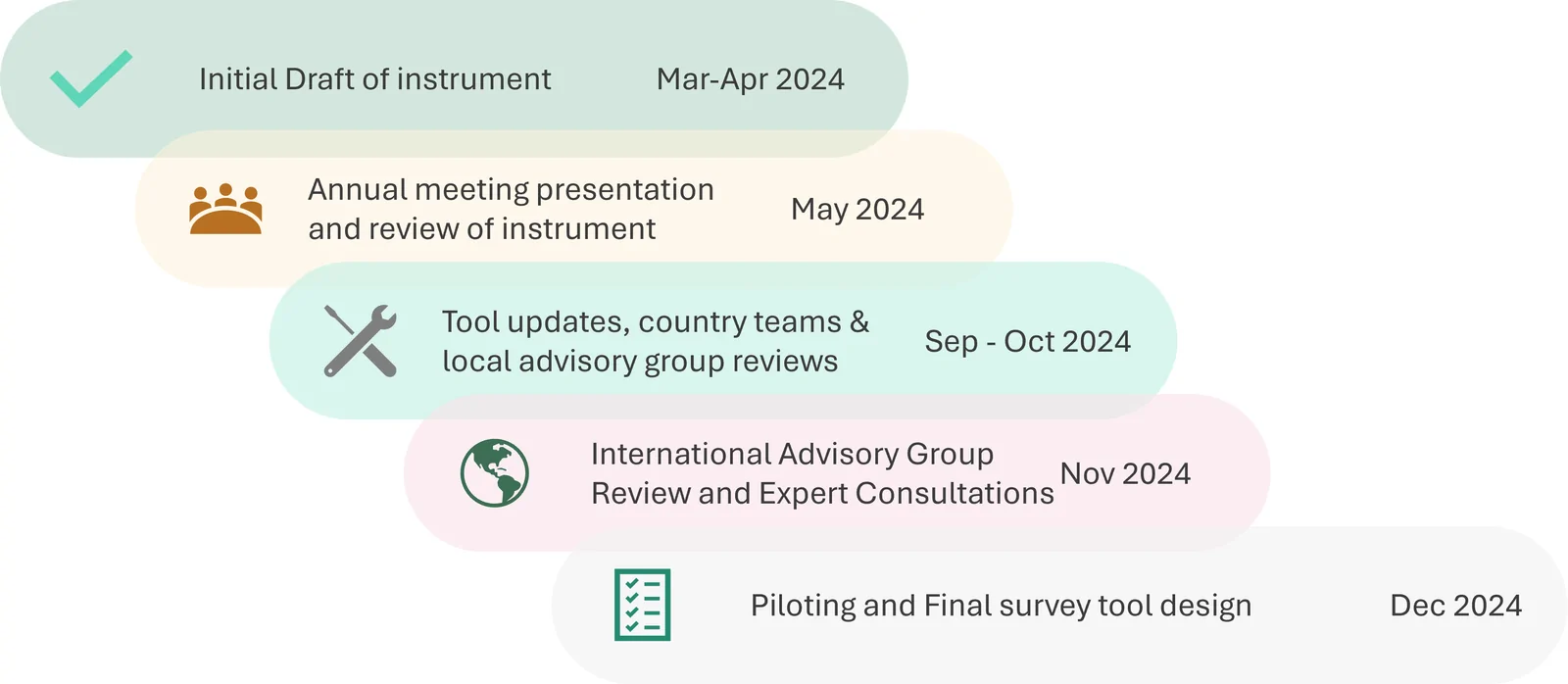
Survey development timeline.

A targeted review of existing survey instruments was undertaken to develop to an overinclusive pool of items and response formats that could be adapted for GEMMS. Following initial draft developments, between May and October 2024 the tool underwent content validation with country teams, in addition to multiple relevant amendments and contextualisation. Critical gaps were identified by the country teams where new items were introduced. Content adequacy and clarity were evaluated through multiple expert reviews in all four country sites. The final survey tool included a total of 216 items across nine sections (online supplemental file 4).

### Generating items for the survey tool

Guided by the conceptual frameworks described above, the survey development process focused on identifying domains and subdomains required to capture migration-related precarity, GV, MH, psychosocial distress, and broader structural determinants across diverse migration contexts.

The initial item pool was generated through a targeted review of existing survey instruments, conceptual frameworks and migration health literature. Constructs and subdomains were identified based on their relevance to the survey objectives and their applicability across the participating country settings.

Wherever possible, items were drawn from widely used and validated population instruments. Where existing instruments were insufficient to capture migration-specific experiences and dimensions of precarity, items were adapted or developed de novo (see figure 2). This was particularly important for domains relating to migration-related adversity, structural vulnerability, governance-related precarity, discrimination and mobility experiences that were not adequately represented in existing measures.

**Figure 2:**
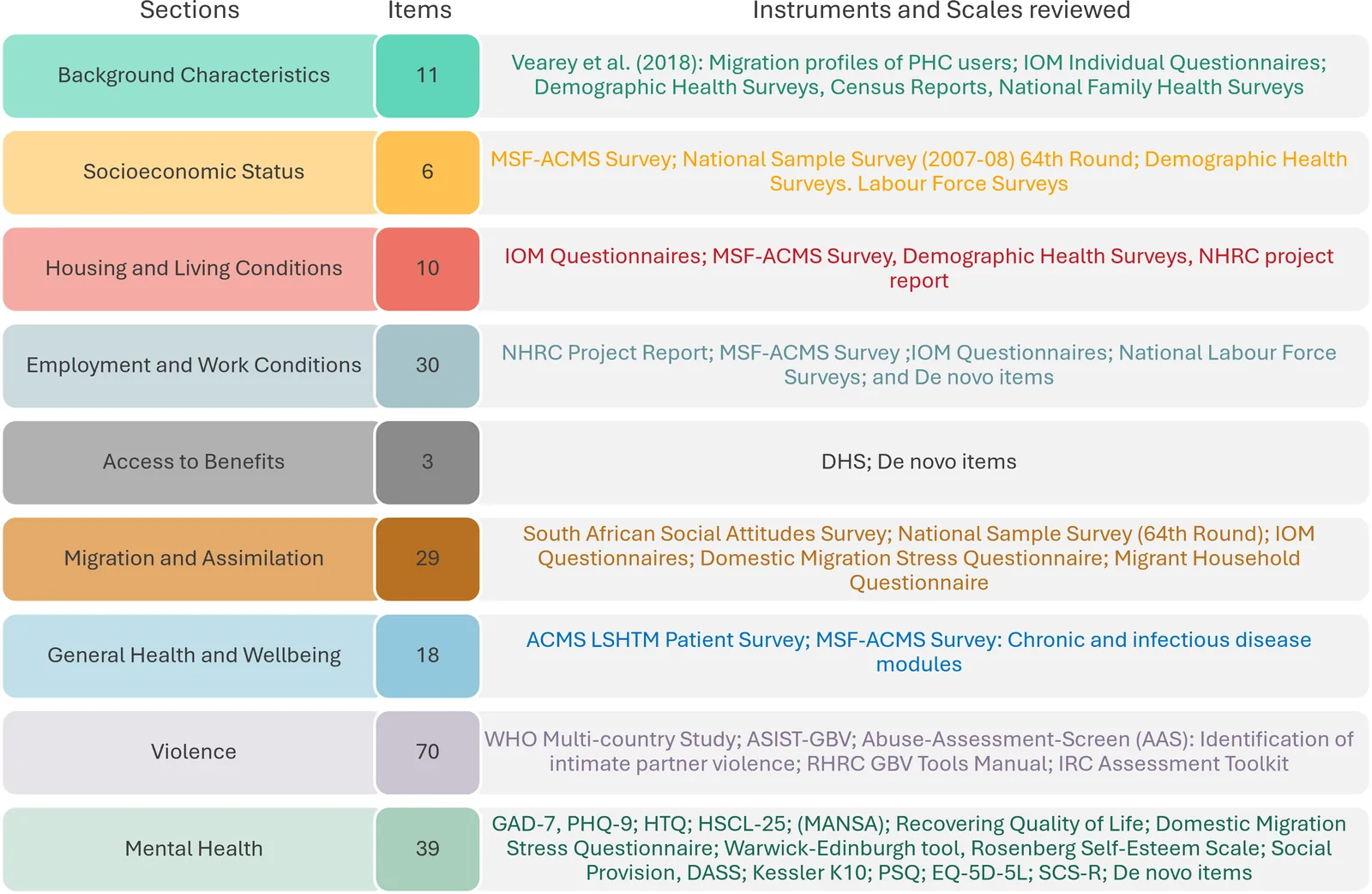
Instruments and scales reviewed to develop the GEMMS survey tool sections. GEMMS, Gendered Violence and Poor Mental Health among Migrants in Precarious Situations. Abbreviations: ASIST-GBV, Assessment Screen to Identify Survivors Toolkit for Gender-Based Violence; DASS, Depression Anxiety Stress Scales; DHS, Demographic and Health Surveys; EQ-5D-5L, EuroQol five-dimension, five-level questionnaire; GAD-7, Generalized Anxiety Disorder seven-item scale; GBV, gender-based violence; HSCL-25, Hopkins Symptom Checklist, 25-item version; HTQ, Harvard Trauma Questionnaire; IOM, International Organization for Migration; IRC, International Rescue Committee; LSHTM, London School of Hygiene & Tropical Medicine; MANSA, Manchester Short Assessment of Quality of Life; MSF-ACMS, Médecins Sans Frontières–African Centre for Migration & Society; NHRC, Nepal Health Research Council; PHC, primary health care; PHQ-9, Patient Health Questionnaire, nine-item version; PSQ, Perceived Stress Questionnaire; RHRC, Reproductive Health Response in Conflict Consortium; SCS-R, Social Connectedness Scale–Revised.

Detailed deliberations on the items, challenges and final decisions, along with the full survey questionnaire and routing logic, have been provided in online supplemental files 1–3.

### Items for the background sections of the survey tool

The background component of the survey tool comprised six sections: background characteristics, socioeconomic status, employment profile, household contexts, benefits access and general health conditions.

The background characteristics were finalised after extensive review of local demographic and health surveys and census reports.^22–24^

The section included measures of financial insecurity, employment conditions, workplace protections, housing circumstances, access to state and humanitarian benefits and general health status. The survey tool was also made flexible for capturing country-specific segregation such as tribal and class classifications. Country-specific response options were incorporated where necessary to improve contextual relevance. For example, the social class categorisation in India was added as a country-specific question only in the otherwise standardised unified tool. Items like education levels required additional multiple iterative consultations with the country teams because of differences in education levels and years across the countries.

Socioeconomic status items included questions on financial insecurity, housing and asset ownership and were adapted to incorporate locally relevant financial savings.

Employment-related items captured questions about contract precarity, payment regularity, work conditions, employment terms, mode of payment, workplace hazards and responses. Finally, a subsection on general health and well-being of migrants was also included, to capture presence of any chronic health conditions, any treatment that they may be undergoing, their usual service providers.

### Items for the migration section of the survey tool

The migration section captured diverse forms of mobility and transience shaping migrants’ lives across India, South Africa, Zimbabwe and Cambodia, as their migration journeys vary considerably depending on national histories, systems and mobility patterns. Migration-related experiences at destination, including service discrimination, and everyday harassment, were measured using items adapted from widely used migrant and population surveys^25–27^ as well as International Organization of Migration (IOM) tools.^28^ De novo items included length of stay, push and pull factors for moving, frequency of return, documentation status, language and cultural fit and experiences of assimilation, discrimination and harassment. Questions mapping the trajectory and temporality of movement were incorporated, allowing the survey to capture heterogeneity of migration processes across the four countries, instead of treating migrants as a single static category. These mobility histories are critical to interpret earlier socioeconomic status questions, employment patterns, documentation status and access to support and state benefits, all influencing the GV and MH profiles of the migrants in question.

### Items for the gender-based violence section of the survey tool

The GV section was designed to estimate the prevalence and patterns of violence encountered by migrants living in precarity. The module captures discrete experiences of violence, coercion and abuse, including in public, at workplace and in intimate settings that migrants may encounter across origin, transit and destination settings. It primarily draws on validated international instruments, most notably the WHO multicountry study questionnaire domains which cover controlling and coercive behaviours, emotional abuse, physical assaults of escalating severity and forced sex.^29^ Adaptations were limited to wording adjustments and contextual examples with respect to migrant labour and mobility settings. No new composite Gender-based violence (GBV) scales were constructed. De novo items were introduced only where standard GBV instruments did not capture migration-specific violence, for example, workplace coercion.

Extensive consultative meetings followed with select GEMMS International Advisory Group (IAG) members with expertise in GV to make the tool contextually relevant. The section also reflects the epidemiological emphasis of the WHO multicountry work on domestic violence that differentiates partner and non-partner perpetrators.^29^ Considering the different nature of violence-related questions to be asked from men, never partnered women and ever partnered women, three sections of violence were developed, with sections using routing logic to ensure that each section is administered appropriately based on gender and relationship status. Violence section for ever partnered women included additional questions on intimate partner violence, detailing controlling behaviours, emotional abuse, graded physical violence and sexual coercion, as well as obstetrical violence.^30^ To preserve comparability, order of common questions in all three sections was kept the same. Additional items were adapted to foreground mobility related vulnerabilities.^31^

The quality of the GV module was assessed through multiple complementary approaches rather than a psychometric evaluation. These included expert review by GBV specialists within the GEMMS IAG and researchers with expertise in gender-based violence, community-based content validation processes, particularly through Local Advisory Group (LAG) consultations, and migrants with lived experiences for assessment of linguistic clarity and contextual relevance.

Particular attention was also given to the routing logic across gender and relationship-status groups to ensure that questions were appropriate to respondents’ lived experiences and minimised unnecessary respondent burden. For example, the men’s violence module focused primarily on experiences of violence, coercion and abuse occurring in migration and employment settings, including interactions with employers and law enforcement authorities. In contrast, the module for ever-partnered women included detailed assessment of intimate partner violence, alongside questions on workplace-related violence where applicable. Separate modules were also developed for never-partnered women to ensure relevance and appropriateness of questioning. Together, these processes ensured that the GV module adequately captured diverse violence experiences across migration contexts while maintaining contextual relevance, respondent safety and feasibility of implementation.

The GV section was therefore designed primarily as an epidemiological event inventory, to capture prevalence, type, setting, frequency, perpetrator and response trajectory, rather than being a reflective scale that would measure a single latent violence construct.

### Items for the mental health section of the survey tool

The starting point for the MH section of the GEMMS survey was to identify common psychological distress, trauma symptoms and social connectedness among precarious migrant populations. This entailed reviewing the most common validated MH instruments and their relevance to the survey’s scope.^32–39^

For core MH symptoms screening, Patient Health Questionnaire (PHQ)-9 and Generalised Anxiety Disorder (GAD)-7 were chosen for depression and anxiety respectively, selected due to their brevity, international and regional validation, alignment with Diagnostic and Statistical Manual of Mental Disorders (DSM) criteria and wide application in LMIC context.^40 41^ The internal consultation and review process was critical in fine-tuning this section.

For example, the Harvard Trauma Questionnaire (HTQ)^42^ was initially selected. However, during GEMMS internal expert review process, it was highlighted that since HTQ focused on torture, war injuries and head trauma, it was less relevant for the GEMMS’ countries’ context and geography. Instead, trauma exposure and symptoms were assessed using the Primary Care Post-Traumatic Stress Disorder (PC-PTSD) screen^39^ as a brief screener that could be administered by non-clinicians in survey conditions. To capture psychosocial dimensions of migrants’ well-being that were not adequately represented by earlier scales, some items from the Revised Social Connectedness Scale-Revised were included,^43^ while some de novo items were introduced to reflect migration-specific stressors.

### Content validation

The content validation process involved three levels of review: (1) international and national expert consultations through the GEMMS IAG, National Advisory Groups and country research teams; (2) multicountry expert review workshops involving migration, gender, mental health and public health specialists; and (3) community-based review through LAGs and Community Reference Groups. Guided by the Communities of Research and Practice (CoRP) approach, the validation process brought together researchers, practitioners and community members to bridge academic theory and lived experience in survey design.^44^ Across these stages, draft versions of the survey tool were reviewed for conceptual relevance, contextual appropriateness, linguistic clarity, cultural resonance, feasibility and respondent burden. Feedback was discussed iteratively by the survey development team and incorporated through successive revisions, centring the voices of those most affected by migration, exclusion and structural marginality.^45^

In Cambodia, content review involved country researchers, Ministry of Health representatives and mental health specialists who assessed contextual relevance, feasibility and appropriateness of proposed measures. In South Africa, iterative reviews with researchers and individuals with lived migration experience informed adaptation to local migration and mobility contexts. In India, expert and community consultations were complemented by a formal Item-level Content Validity Index (I-CVI) assessment with 10 LAG members. Thus, while content validation was undertaken across all the participating countries, structured quantitative assessment of content validity was conducted only in India and contributed to the broader multi-country validation process.

In India, content validation of 24 items was undertaken with LAG members with lived experiences of working with migrant and marginalised communities. This constituted the only site where a structured I-CVI assessment was conducted. The items were selected from each section based on their conceptual importance and to provide sufficient coverage to identify patterns in language use and comprehension.^46^ A structured content validation template was developed, allowing the participants to rate the items on Relevance, Clarity and Comprehension on a four-point response scale. Item-level content validity was subsequently calculated. Of the 24 items, 16 items achieved a content validity index ≥0.78, which meets the widely accepted threshold for excellent content validity.^47^ Items with I-CVI values below 0.78 were not automatically removed. Instead, questions with lower scores were predominantly due to comprehension challenges and contextual interpretation rather than a lack of conceptual relevance. These questions were revised through collaborative follow-up discussion with LAG members, and based on these discussions, wording was simplified, colloquial expressions were incorporated and additional probes or examples were introduced where appropriate. Consequently, low-scoring items were retained in revised form rather than deleted. Details for language simplification and content validation scores in India are furnished in online supplemental file 2.

To improve feasibility, multiple skip logics were also implemented to reduce reporting burden and fatigue on respondents for questions irrelevant to them (online supplemental file 2).

### Pilot testing and survey tool refinement

The final version of the unified modular survey tool, with 216 items across nine sections, was formally signed off by each country team prior to its digitisation (online supplemental file 4) before proceeding with its piloting.

The pilot study was conducted between November and December 2024 in South Africa, India and Cambodia. The sites were selected based on prior participatory mapping and field reconnaissance that identified them as densely populated informal migrant settlements with high levels of spatial precarity, occupational risk and limited access to basic services. Multistaged sampling was done and piloting was guided by detailed field protocols for community-based surveying, including detailed training on adversity and risk management, distress management, anonymity and confidentiality. Complete training for survey teams across all sites, including detailed ethics training, was completed in advance of the pilot testing. An average of 60–70 interviews were conducted in each country site during the pilot phase, yielding a total of 198 interviews (see table 1). This number represents roughly 10% of the intended full sample size calculated, a proportion considered reasonable for piloting and to assess reliability and preliminary face and content validity.^48^ The eligibility screening for pilot participants included: (1) being 18 years or older; and (2) having moved to the current site within the last 10 years. Pilot testing in all three sites started by 15 November 2024 and completed in 3 weeks. Pilot sites were earmarked to be excluded in the final survey sampling.

**Table 1:** Demographics of pilot participants

Country	Gender	Marital status		Total
		Never partnered	Ever partnered	
Cambodia	Female	3	33	36
	Male	8	24	32
	Other	0	2	2
	Total	11	59	70
India	Female	0	33	33
	Male	21	9	30
	Total	21	42	63
South Africa	Female	3	42	45
	Male	1	17	18
	Other	0	2	2
	Total	4	61	65
Total	Female	6	108	114
	Male	30	50	80
	Other	0	4	4
		36	162	198

The pilot study was also used to assess the operational feasibility, acceptability and ethical administration of the survey tool across diverse migrant settings. Given the breadth of sensitive topics included in the survey, particular attention was paid to participant safety, respondent burden, privacy, distress management, and administration procedures during pilot implementation.

The GEMMS project operated under a common ethics, safeguarding and distress management framework developed in accordance with NIHR requirements and implemented across all participating countries. All researchers and field staff involved in piloting completed NIHR-certified ethics, safeguarding and distress management training. In addition to project-wide training, senior research team members in each country conducted locally contextualised training sessions in relevant languages to ensure consistent implementation before data collection commenced. These trainings covered informed consent procedures, confidentiality, anonymity, interviewing on sensitive topics, distress recognition and management, participant safety, and referral procedures.

Interviews were conducted in private settings wherever feasible. However, maintaining complete privacy was not always possible in precarious settlements due to overcrowded living conditions and community curiosity regarding research activities. Field teams were therefore trained to assess privacy continuously throughout the interview. In situations where privacy was temporarily compromised, interviewers used pre-agreed neutral (dummy) questions until privacy could be re-established. Participants were informed that they could decline to answer any question, skip individual items or discontinue participation at any stage without consequence. Automated routing and skip logic were incorporated into the digitised survey tool to minimise unnecessary exposure to sensitive questions and reduce respondent burden.

A common distress management and safeguarding protocol was implemented across all participating countries. Interviewers were trained to recognise signs of distress, discomfort, fatigue or potential risk, and to pause, reschedule or discontinue interviews where necessary, prioritising participant well-being over survey completion. Participants were regularly reminded that they could skip questions, take breaks or withdraw from the survey at any point without consequence.

Where participants disclosed experiences of violence, severe distress or other situations requiring support, interviewers followed predefined safeguarding procedures and engaged in supportive discussion regarding available sources of assistance. Participants were provided with information on locally available services, including mental health support, gender-based violence services, social welfare programmes and relevant government or community-based organisations. Country-specific referral pathways and contact information were developed and made available across study sites to facilitate access to appropriate support where required.

Support mechanisms were further strengthened through community engagement structures established within the GEMMS project. For example, in India, community field guides and LAG members, many of whom had longstanding links with local organisations working on gender-based violence, mental health, housing insecurity and migrant welfare, assisted in connecting participants to appropriate sources of support where needed. Similar community-based support mechanisms and referral pathways were adapted within other partnering countries’ contexts to strengthen ethical engagement and participant safeguarding during field implementation.

Feedback from the pilot implementation primarily focused on enabling the research teams to identify situations where additional routing conditions were required to account for the flow of the digitised questionnaire. There was limited feedback regarding item content, language or comprehension, as these aspects had already been extensively reviewed and refined through expert consultations, community validation processes and LAG inputs. The resulting refinements were incorporated into the final survey tool prior to multicountry implementation. The administration of 198 pilot interviews across three country sites provided critical inputs on operational feasibility and evidence of acceptability of the survey tool in diverse migrant settings.

### Maintaining cross-country comparability in a uniform standardised tool

While the tool was designed as a modular tool, the intent was to have a shared core structure that allowed cross-country comparisons, while also allowing limited contextual adaptation so that country-level granularities are not ignored. This was done through four steps. First, a single unified master survey tool was developed that all the country teams worked on, organised around common domains like background, socioeconomic status, employment status, migration history, GV and MH, etc. Second, based on desired analysis and cross-country comparisons, core constructs, items, response structure and recall periods were standardised. These were the core standardised items administered across all sites with the same construct meaning and response options. Third, where adaptation was necessary, change was restricted and multiply reviewed to ensure that the underlying construct remains relevant. These included two types of items: (a) locally adapted items, where wording or translations were modified to improve comprehension while preserving the measurement, for example, the state benefits schemes; and (2) country-specific items included to capture locally salient classifications, migration regimes, ethnicity, type of savings, for example, migration categories, additional social groups for India. Fourth and final, all country-specific additions were clearly marked and not treated as comparable pooled indicators unless they could be harmonised analytically.

This approach enabled the survey to retain a common comparative architecture while avoiding false equivalents across diverse migration settings. All the local adaptations were elaborated in the survey manual prepared for the survey teams (see table 2 below).

**Table 2:** Approaches to standardise survey tool

Survey module	Elements standardised across countries	Items adapted linguistically, culturally	De-novo items
Background	Gender, age, education, relationship status, household composition	Wordings adapted to local language/culture	Social group categories for India
Socioeconomic status and housing	Food insecurity, housing conditions	Assets, environmental risks	Saving types
Employment and labour conditions	Employment status, occupation, work and contract type, payment modality, workplace hazards,	Occupational categories, state benefit schemes	State benefit schemes
Migration history	Migration pattern, length of stay, reasons for migration, documentation	Migration routes	–
Gendered violence	Common event-based inventory	Wording adapted for safety, cultural comprehension.	–
Mental health	Standardised items, and additional psychosocial and migration stress related items	Local prompts, local idioms of distress during administration	–

This classification ensured that not all differences be treated as measurement errors. Some differences were retained since they reflected meaningful variation at the country level, for example, social class, or migration routes. However, none of these items were intended to be interpreted as comparable indicators or pooled in composite indices. Country-specific categories will only be used to support in-country analysis and interpretation.

### Factor analysis

For selected adapted and de novo multi-item construct-oriented domains, the internal coherence was assessed through exploratory factor analysis (EFA) and only to assess internal coherence of theoretically grouped items that lacked prior validation. These were migration-related adversity items and non-clinical mental health items, like living in fear. Established clinical screening instruments like GAD-7, PHQ-9 and PC-PTSD were not included since they already have well established validation data and their established structures were retained. GV related items were also not factor analysed since the module was designed as an event-based inventory and not a reflective latent scale. The module captured heterogeneous violence events across different perpetrators in different settings with different temporalities, operationalised through event exposure profiles, type-specific violence measures, perpetrator-specific experiences, cumulative violence indicators and response or help-seeking cascades. For such a design, EFA can be misleading since some violence events occur rarely but are crucial, some occur together while others happen alone, some are routing-dependent, some are conditional on gender and partnership status, some are about exposure, some about frequency, then perpetrator and help-seeking. Accordingly, the pilot data of the GV section was reviewed for item comprehension, acceptability, sequencing and response distribution.

EFA and reliability analyses, on the other hand, were intended to assess the internal coherence of selected item sets that were either newly developed or adapted for the GEMMS survey tool.^49^

In this survey tool, EFA was used pragmatically and did not constitute psychometric validation of the entire instrument. No attempt has been made to claim full psychometric validation of the entire GEMMS survey tool.

EFA was done in SPSS using Principal Axis Factoring with Promax rotation and Kaiser normalisation, since the constructs were (expected to be) conceptually related. All cases were retained for analysis. Factorability was assessed using the Kaiser-Meyer-Olkin (KMO) measure of sampling adequacy, and Bartlett’s test of sphericity. The number of factors were determined using eigenvalues >1, scree plot and the interpretability criterion.

Items were reviewed for retention based on primary pattern loading of >0.40, extraction communalities cross-loadings (using pattern matrix) and conceptual coherence with the emerging factor. Any item with substantial secondary loadings, weak primary loadings, low extraction communalities or poor conceptual fit were assessed for removal or retention. Internal consistency of the retained factors was assessed using Cronbach’s alpha, corrected item-total correlations, alpha-if-item-deleted statistics and inter-item correlations.

## Results

### Construct coherence for selected item sets and final refinement

A total of 198 migrants participated in the pilot survey, as depicted in table 1 below, with 57.6% females and 42.4% males. There were 70 survey respondents from Cambodia, 63 from India and 65 from South Africa.

Consistent with the modular design of the GEMMS survey tool, the post-pilot assessment strategies varied by sections. Where the survey included established clinical screeners, their structures were retained in validated forms.

For the migration-related adversity items set, the initial model was estimated with 13 items. Principal Axis Factoring with Promax rotation was used and factorability supported by KMO=0.852 and statistically significant Bartlett’s test of sphericity χ²(66) = 888.892, p<0.001 (EFA_mig_ round 1 in table 3). This indicated that the correlation matrix was suitable for factor analysis. Items were also reviewed for communalities, pattern loadings, cross-loadings and conceptual coherence. Items with low communalities or limited conceptual coherence were removed (n=5), and the model was re-estimated (EFA_mig_ round 2 in table 3).

**Table 3:** EFA_mig_ and reliability assessment for migration-related items

EFA_mig_	Items retained	KMO measure of sampling adequacy	Bartlett’s test of sphericity	Low extraction communalities (<0.40)
EFA_mig_ Round 1	13 entered	0.852 - Meritorious	888.89, p<0.001 - Factorable correlations	Access to quality health, inability to express self, treated with suspicion, receive poorer care
EFA_mig_ Round 2	8 retained	0.833 - Very good	643.38, p<0.001 - Factorable correlations	Final 2-factor solution retained
Factors extracted from EFA_mig_ Round 2				
Factor correlation r=0.596, moderate	Theme	Factor 1 - targeted structural harm	Factor 2 - sociocultural marginalisation	
	Items retained	(Items # 277,278, 279, 280) Threat to life, threat of arrest, threat of torture and fear of being expelled.	(Items # 282, 283, 285, 287) Inability to enjoy culture, practice religion, treated with less respect and called names in public	
	Pattern loadings	0.59–0.80 - Strong and clean loadings	0.43–0.72 - Two strong cultural items and two moderate but cleanly loading social items with theoretical coherence.	
Reliability of final factors	Cronbach’s alpha	0.834 - Very good	0.763 - Acceptable	
	Inter-Item correlation	0.48–0.61 - Strong and positive	0.32–0.63 - Within ideal range	
	Corrected item - total correlations	0.60–0.74 - Strong	0.55–0.58 - Strong	
	Alpha if items deleted	0.755–0.819 - Removing any item reduces reliability	0.700–0.716 - Removing any item reduces reliability	
	Conclusion	Theoretically coherent and internally consistent	Reliable and conceptually consistent	

EFA_mig_, migration-related exploratory factor analysis; KMO, Kaiser-Meyer-Olkin.

The final migration-related EFA included eight items, where the effective sample size was 198 cases, with no missing data. The final model exhibited strong factorability, with KMO=0.833 and Bartlett’s test of sphericity χ²(28) = 643.378, p<0.001.

Two factors were extracted from EFA_mig_ 2, with eigenvalues greater than one, and were thus retained. This solution explained 52.954% of the extracted variance. Factor 1, labelled ‘targeted structural harm’ included threat to life, threat of arrest, threat of torture and fear of being expelled, with strong pattern loadings ranging from 0.59 to 0.80. Factor 2 labelled ‘socio-cultural marginalisation’ included inability to enjoy culture, practice religion, treated with less respect and being called names in public, with pattern loadings ranging from 0.43 to 0.72. The factor correlation was moderate with r=0.596, supporting the use of oblique rotation. Internal consistency was good for structural harm at Cronbach’s α=0.834, and acceptable for socio-cultural marginalisation, at α=0.763.

A similar process was undertaken for the mental health-related item set (table 4). Seventeen adapted and de novo items were initially included into EFA using the same extraction and rotation approaches. Factorability was supported by KMO=0.856 and Bartlett’s test of sphericity χ²(136) = 1559.212, p<0.001. However, two factors were removed due to low extraction communalities (not feeling involved with people extraction=0.257, and missing family/friends back home extraction=0.207). ‘Did not expect it to be so difficult’ had marginal extraction=0.383 but was retained due to its conceptual importance. The three factors were retained based on eigenvalues>1, scree plot and theoretical interpretability. This three factor solution explained 52.215% of the extracted variance. The factors were labelled ‘social exclusion’, ‘belonging and social integration’ and ‘migration stress’. Social exclusion included feeling isolated, disconnected, struggling with customers, not feeling belonging, being treated unfairly, looked down up, hard to make friends and feeling like an outsider, with strong pattern loadings ranging from 0.62 to 0.82. Belonging and social integration included feeling close to people, feeling belonging, feeling fitting in and relating to others, with pattern loadings from 0.64 to 0.85. Finally, migration stress included items capturing earning money harder than expected, things not going as planned, life not as imagined, with pattern loadings 0.60 to 0.99. Factor correlations ranged from weak to moderate. Internal consistency was excellent for social exclusion (Cronbach’s α=0.844), and good for belonging (Cronbach’s α=0.795) and migration stress (Cronbach’s α=0.795).

**Table 4:** EFA_mental_ and reliability for MH-related items

EFA	Items retained	KMO measure of sampling adequacy	Bartlett’s test of sphericity	Low extraction communalities (<0.40)
EFA_mental_ Round 1	17 entered	0.856 - Meritorious	p<0.001 - Factorable correlations	Three factors extracted. Two items had low extraction communalities and were removed (V794 and V805)
Factors extracted				
Factor correlation r=0.013 to 0.396	Theme	Factor 1: social exclusion	Factor 2: belonging and social integration	Factor 3: migration stress
	Items retained	(Items # 797, 798, 799, 800, 801, 802, 803, 804) Feeling isolated, disconnected, struggling with customers, not feeling belonging, being treated unfairly, looked down up, hard to make friends and feeling like an outsider	(Items # 792, 793, 795, 796) Feeling close to people, feeling belonging, feeling fitting in and relating to others	(Items # 806, 807, 808) Earning money harder than expected, things not going as planned, life not as imagined
	Pattern loadings	0.62–0.82 - Strong and clean	0.64–0.85 - Strong	0.60–0.99 - Strong
Reliability of final factors	Cronbach’s Alpha	0.895 - Excellent	0.844 - Very good for four items	0.795 - Good for three items
	Inter-Item correlation	0.377–0.734 - Strong without redundancy	0.458–0.661 - Moderate to strong	0.416–0.688 - Moderate to strong
	Corrected item -total correlations	0.621–0.804 - Strong	0.615–0.751 - Good	0.548–0.757 - Strong
	Alpha if items deleted	0.877–0.891 - Removing any item reduces reliability	0.769–0.831 - Removing any item reduces reliability	0.588–0.815 - Removing 806 economic hardship may improve alpha but reduce conceptual breadth. Hence retaining
	Conclusion	Psychometrically and theoretically coherent and internally consistent	High quality scale with strong cohesion	Good reliability and conceptual coherence

EFA, exploratory factor analysis; EFA_mental_, mental health-related EFA; KMO, Kaiser-Meyer-Olkin; MH, mental health.

These findings supported the internal coherence and reliability for the selected adapted and de novo migration-related and psychosocial items set. They should not be interpreted as a full psychometric validation of the GEMMS survey tool.

The findings from EFA should be interpreted as preliminary evidence rather than definitive psychometric validation. The sample size for this pilot validation study was modest (n=198) and drawn from three countries met the thresholds required for EFA. No missing data were present in the analysed items; all 198 items were retained for analysis. All the retained items in the final subscales are theoretically strong, showing high-factor loadings and were therefore retained. The extracted factors were interpretable, aligned with theoretical expectations from the migration literature and supported by clear conceptual domains in the survey design. Due to the multicountry and multilingual nature of the pilot data, the factor solution need to be interpreted with caution. Additionally, the pilot was not powered to assess measurement invariance across countries, languages, gender groups or other subpopulations. A number of item retention decisions were made based on statistical performance and theoretical relevance. These findings do not test syndemic co-occurrence, causality of pathways or intersectional differences. The final GEMMS survey sample should do those tests in addition to confirming the factor structure, assessing cross-country measurement equivalence, examining criterion validity and testing whether the derived factors function similarly across different migrant groups.

### Methodological trade-offs

In this entire process, several key methodological trade-offs were made, which shaped the survey design and item selection across domains, as illustrated in figure 3, with additional details provided in online supplemental file 1.

**Figure 3:**
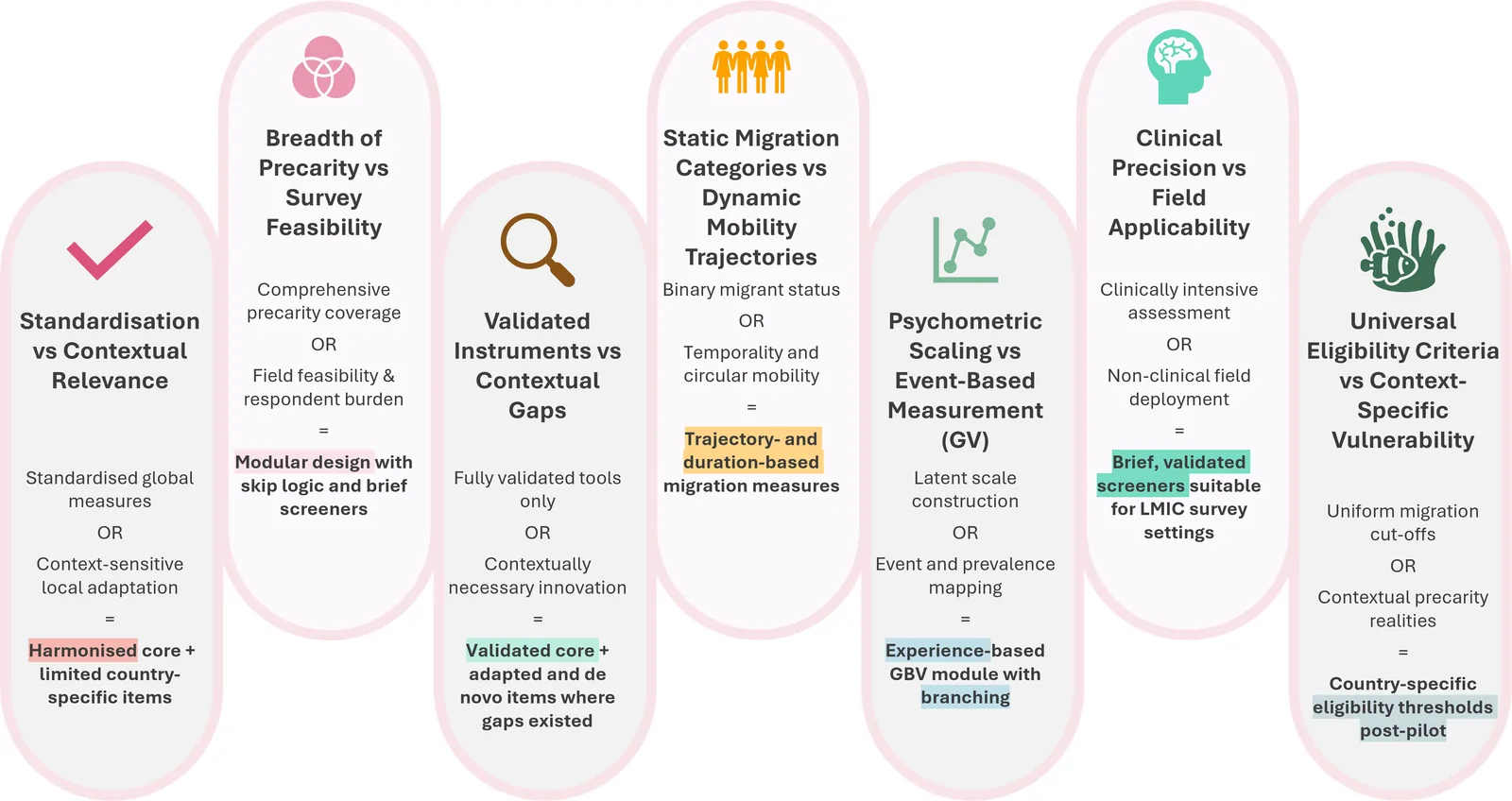
Main design trade-offs shaping the GEMMS survey tool. GEMMS, Gendered Violence and Poor Mental Health among Migrants in Precarious Situations; GBV, gender-based violence; GV, gendered violence; LMIC, low- and middle-income country.

Since the GV items were incorporated as part of an event-based epidemiological module rather than a latent scale, factor-analytical assessment was not considered the appropriate first-stage approach. Later these items will require further evaluation in survey data analyses, including examination of endorsement patterns, cross-country functioning and criterion relevance.

## Discussion

Factor-analytical assessment of selected items in GEMMS survey tool yielded five interpretable and internally coherent factors across the migration and MH domains. For migration-related experiences, two factors emerged with excellent psychometric properties: Factor 1 (Targeted structural harm) captured direct threats, including arrest, torture and expulsion, with strong factor loadings (0.59–0.80), while Factor 2 (Sociocultural marginalisation) reflected cultural and social exclusion experiences with loadings ranging from 0.43 to 0.72. For MH, three distinct factors demonstrated strong internal consistency: Factor 1 (Social exclusion) with loadings of 0.62–0.82, Factor 2 (Belonging) with loadings of 0.64–0.85, and Factor 3 (Migration stress) with loadings of 0.60–0.99. Within the constraints of this pilot sample, these reliability coefficients meet or exceed widely accepted thresholds (Cronbach’s alpha≥0.70 for acceptable, ≥0.80 for good, ≥0.90 for excellent), with most inter-item correlations falling within optimal ranges (0.30–0.70), indicating strong internal consistency without redundancy.^50^ These findings should be interpreted as evidence for internal coherence and not a full psychometric validation, which will be examined in the full survey sample, along with measurement invariance testing of the full survey data and formal analysis of syndemic and intersectional relationships.

The internal consistency of these new factors falls within the ranges reported for established instruments, although comparability cannot be assumed without further validation with full survey data of migrant populations. A recent systematic review and meta-analysis examining MH measurement among international migrant workers^51^ identified that the most commonly validated self-report questionnaires include the PHQ-9 for depression, GAD-7 for anxiety and the Migrant Stress Inventory for migration-specific stress. While these tools show acceptable reliability (Cronbach’s alpha typically 0.75–0.89 for PHQ-9 and GAD-7 across diverse migrant populations), they primarily capture clinical symptoms aligned with psychiatric diagnostic criteria. The new migration-specific MH factors (social exclusion, belonging and migration stress) were designed to capture psychosocial dimensions of MH burden that are particularly salient for precarious migrants but inadequately measured by symptom checklists alone. This multidimensionality is particularly important for intervention design. Additionally, while the PHQ-9 and GAD-7 (also included in the GEMMS tool) indicate symptom severity requiring clinical attention, the new migration-specific subscales (structural harm and sociocultural marginalisation) identify specific social and structural determinants such as workplace discrimination, cultural integration and discrimination, that helps understand the pathways to these MH problems and identify targeted programmatic responses beyond MH services alone. Whether these subscales perform comparability to established instruments across diverse migrant populations remains to be confirmed in the full survey sample.

This paper specifically makes two key contributions to migrant health research in LMICs. First, it shows how intersectionality and syndemicity can be used as a design imperative, and these frameworks translated into a modular survey design that integrates pre-validated and newly developed MH subscales with event-based GV inventory and multidimensional precarity. This paper does not test syndemic relationships between GV or MH, or precarity, but establishes a survey architecture through which such interacting burdens and the structural conditions with contextual granularities may be examined in future.

Second, the tool provides a basis for future intersectional analysis by systematically capturing social locations (gender, migrant status, class, ethnicity), migration-related conditions, precarity dimensions alongside MH and GV outcomes. However, intersectional analysis itself has not been undertaken in this paper.

Additionally, the CoRP approach embedded in our development process demonstrates a cascading, bi-directional, participatory and context-responsive approach to research tools’ development in LMIC settings. By involving international advisors, country research teams, LAG members with lived experiences, the item relevance, acceptability, linguistic clarity and contextual fit could be refined, ensuring ground-up feedback with cross-country harmonisation across four LMICs, and creating a replicable blueprint for participatory research design.

Future analyses will need to examine how specific combinations of gender, migration status, class, ethnicity or other social locations shape exposure to GV and MH burden and access to support across different country settings.

## Conclusion

The GEMMS survey tool advances structurally informed measurement for migrant health research by integrating domains often studied in isolation, such as GV, MH, psychosocial well-being and multidimensional precarity, within a single, contextually grounded instrument. Informed by syndemicity and intersectionality perspectives, the tool has been designed to capture not just MH burden and GV exposure, but also the structural and migration-related factors that drive these.

This paper demonstrates conceptual development, participatory refinement grounded in CoRP principles, pilot feasibility and preliminary validation evidence for selected components of the GEMMS survey tool. The EFA provides early evidence of internal coherence for selected adapted and de novo items, but does not constitute full psychometric validation of the instrument.

The survey tool’s broader analytical potential will be tested in future work, using the full survey sample to examine the co-occurrence and intersecting pathways of GV, MH and precarity among migrant populations in LMICs. Future analyses should also assess intersectional differences across migrant subgroups; test measurement invariances across countries and languages, assess predictive validity and evaluate the validity of the newly adapted components. In this case, the GEMMS survey tool provides a foundation to move beyond prevalence estimates towards more exploratory analysis of migrant health inequities, and undertake future syndemic and intersectional analysis, as migration regimes and labour contexts evolve.

## Supplementary material

10.1136/bmjopen-2026-120912 online supplemental file 1

10.1136/bmjopen-2026-120912 online supplemental file 2

## Data Availability

Data are available upon reasonable request.
