# Supplementary material for: Developing a survey tool to assess gendered violence and mental health burden in migrants living in precarity
Source: BMJ Open. 2026 Sep 30;16(9):e120912. doi: 10.1136/bmjopen-2026-120912 (PMC13630092; doi:10.1136/bmjopen-2026-120912)
Supplement: Supplementary file 1 [file bmjopen-2026-120912.reviewer_comments.pdf]

PEER REVIEW HISTORY

BMJ Open publishes all reviews undertaken for accepted manuscripts. Reviewers are asked to complete a checklist review form and are provided with free text boxes to elaborate on their assessment. These free text comments are reproduced below.

ARTICLE DETAILS

Title (Provisional)

Developing a survey tool to assess gendered violence and mental health burden in migrants living in precarity

Authors

Adsul, Neha; Aziz, Roomi; Sivakami,, Muthusamy; Bhui, Kamaldeep; Kapilashrami, Anuj; GEMMS Global Health Research Group, NIHR-funded

---

VERSION 1 - REVIEW

**Reviewer**	**1**
**Name**	**Schoenmakers, Birgitte**
**Affiliation**	**KU Leuven, Public Health and Primary Care**
**Date**	**22-May-2026**
**COI**	**None**

Overall, this manuscript addresses a highly relevant and important topic. However, there are concerns regarding its internal coherence and methodological transparency. Across sections, there appears to be a lack of alignment between the study aims, methods, and reported results.

First, the methods section does not provide sufficient detail to ensure reproducibility. Key processes, such as the development of the tool items and the content validation procedure, are insufficiently described, and no clear methodological framework is specified (e.g. Delphi method, Content Validity Index approach).

Second, there are inconsistencies between the methods and results sections. For example, results referring to a pilot study are presented without a corresponding description in the methods. Similarly, the justification of the sample size is based on criteria for pilot studies assessing face and content validity, whereas the stated aim is to assess construct validity. This raises concerns, as construct validity typically requires different methodological approaches and larger sample sizes.

Third, from a structural perspective, the manuscript at times conflates methods, results, and discussion. Certain sections appear to mix methodological description and interpretation

without clearly presenting the underlying results, making it difficult to assess the evidence supporting the conclusions.

Finally, the reporting of the entire tool development process, including item generation, validation, and testing, appears somewhat fragmented and difficult to follow. Given the complexity of these steps, the current presentation may be too dense or ambitious for a single paper, and could benefit from clearer structuring or even consideration of separating phases of the development process.

Taken together, these issues limit the transparency and interpretability of the study. I recommend revising the manuscript to ensure (1) a clear and detailed description of all methodological steps, (2) full alignment between aims, methods, and results, (3) a clearer separation between results and their interpretation, and (4) a more structured presentation of the tool development process.

**Reviewer**	**2**
**Name**	**Yamashita, Tadashi**
**Affiliation**	**Kyoto University, Graduate School of Medicine**
**Date**	**26-May-2026**
**COI**	**None**

Thank you for the opportunity to review this manuscript. This paper describes the development and early validation of the GEMMS modular survey tool, which aims to assess gendered violence, mental health burden, and migration-related precarity among migrants living in precarious conditions. The topic is important and timely, particularly given the limited availability of survey instruments that can capture the structural and contextual conditions shaping migrants' experiences of violence and poor mental health.

I found the manuscript ambitious and potentially valuable. Its main strengths are the participatory and multi-country development process, the explicit attention to structural precarity, and the attempt to bring together gendered violence, mental health, migration trajectories, and service access within one survey architecture. At the same time, I think the manuscript would benefit from further clarification in several areas before publication. In particular, the distinction between developing a comprehensive modular tool and providing early validation evidence for selected components needs to be made more consistently throughout the paper.

Major comments

1. Please clarify the extent of validation being claimed.

The manuscript presents the GEMMS tool as a context-responsive and structurally informed survey instrument. This is a reasonable and important contribution. However, the current

empirical validation appears to apply only to selected newly developed or adapted items, rather than to the full 216-item tool. Although the authors do acknowledge that full psychometric validation is still needed, some parts of the abstract, results, and conclusion seem to imply a stronger level of validation than the data can support. I would suggest using more cautious wording throughout, such as “early validation,” “pilot validation,” or “initial validation of selected components.”

2. The exploratory factor analysis needs fuller reporting.

The factor analysis is one of the main empirical findings of the paper, but the current description is not detailed enough for readers to judge the robustness of the results. Please provide more information on the number of items entered into each analysis, the effective sample size after missing data and skip patterns, extraction method, rotation method, criteria for deciding the number of factors, loading thresholds, and how cross-loadings were handled. It would also be useful to report whether factorability was assessed, for example using KMO and Bartlett’s test. Given the relatively small pilot sample and the multi-country nature of the data, the limitations of the factor solution should be discussed more explicitly.

3. Please explain more clearly how cross-country comparability was maintained.

One of the most interesting features of the tool is the balance between standardisation and local adaptation. However, it is not always clear which items were identical across sites, which were adapted linguistically or culturally, and which were country-specific. This distinction is important because the tool is intended to support comparison across different migrant populations and settings. A clearer explanation, preferably with a summary table, would help readers understand how comparability was preserved despite necessary contextualisation.

4. The rationale for treating the gendered violence module as an event inventory should be strengthened in the main text.

I agree with the authors’ decision not to treat the gendered violence section as a reflective psychometric scale. The module appears to be designed to capture discrete events, perpetrators, settings, frequency, and help-seeking responses, rather than a single latent construct. This is methodologically appropriate. However, this point should be stated more clearly in the main manuscript. The authors should also describe how the quality of this section was assessed, for example through content validity, acceptability, routing logic, ethical safety, and alignment with established gender-based violence assessment frameworks.

5. The content validation process should be described more systematically.

The participatory development process is a major strength of the study, but the reporting of content validation could be clearer. Please provide more detail on who participated in each stage, how many people were involved, how relevance and comprehensibility were assessed, and how the final decisions were made. In particular, some items appear to have

relatively low item-level CVI values. It would be helpful to explain whether these items were revised, retained, removed, or further discussed, and what decision rules were used.

6. Feasibility and respondent burden require more attention.

The final tool contains 216 items and includes sensitive topics such as violence, mental health, trauma, documentation insecurity, and suicidal ideation. This raises important practical and ethical questions. Please report, if available, the approximate duration of administration, completion rates, interviewer feedback, participant burden, and any difficulties observed during the pilot. This information is especially important because the tool is intended for migrants living in precarious circumstances.

7. Ethical safeguards should be described in greater detail.

Because the survey includes highly sensitive questions, the manuscript should provide more detail on the ethical procedures used during data collection. In particular, please describe how privacy was ensured during interviews, how interviewers were trained, what distress protocols were in place, how disclosures of violence or self-harm risk were managed, and what referral pathways were available. These details are essential for assessing the practical and ethical robustness of the tool.

8. Please separate current findings from future analytic potential.

The manuscript rightly notes that the GEMMS tool may support future syndemic and intersectional analyses. However, these future possibilities should be distinguished more clearly from what has actually been demonstrated in the present paper. At present, the manuscript demonstrates the development process and early validation evidence for selected components, rather than the full analytic potential of the tool.

Minor comments

1. The abstract should state more clearly that this is a development and early validation study of selected components of the tool, rather than a full validation of the entire instrument.

2. A table summarising the nine sections of the tool, the number of items in each section, the item sources, and the type of validation conducted would improve readability.

3. Please provide more detail on the pilot sample, including the number of participants by country and key demographic characteristics.

4. Please explain how missing data were handled in the pilot analyses, especially in the factor analysis.

5. Key terms such as “gendered violence,” “precarity,” “governmentality-based precarity,” “structural harm,” and “sociocultural marginalisation” should be defined clearly and used consistently.

6. Some sections of the background and discussion repeat similar points about structural vulnerability, syndemics, and intersectionality. The manuscript would benefit from some tightening.

7. Several sentences are long and difficult to follow. Careful language editing would improve readability.

8. Please use a consistent name for the instrument throughout the manuscript.

VERSION 1 - AUTHOR RESPONSE

Section	Comments	Response and Changes made	Section and/or line numbers
**Abstract**	**Minor Comment 1**. The abstract should state more clearly that this is a development and early validation study of selected components of the tool, rather than a full validation of the entire instrument.	Thank you - we have revised the abstract to state explicitly that this is an early validation study of selected components of the tool, not a full validation of the entire GEMMS survey instrument. The aims and results section of the abstract (and the corresponding sections in the body) are updated to reflect this distinction	See revised abstract
**Overall**	**Reviewer 2: Comment 8:** Please separate current findings from future analytic potential. The manuscript rightly notes that the GEMMS tool may support future syndemic and intersectional analyses. However, these future possibilities should be distinguished more clearly from what has actually been demonstrated in the present paper. At present, the manuscript demonstrates the development process and early validation evidence for selected components, rather than the full analytic potential of the tool.	Thank you. We do agree and have accordingly revised the abstract, strengths and limitations, discussion and conclusion sectors to avoid this implication that syndemic or intersectional relationships have already been tested empirically.	Edits made throughout the manuscript.
	**Reviewer 2: Comment 1:** Please clarify the extent of validation being claimed. The manuscript presents the GEMMS tool as a context-responsive and structurally informed survey instrument. However, the current empirical validation appears to apply only to selected newly developed or adapted items rather than the full 216-item tool. Some sections imply a stronger level of validation than the data	We thank the reviewer for this important observation and agree that the scope of validation should be more explicitly stated. We wish to clarify that the GEMMS survey instrument itself is not a pilot tool. Rather, the pilot study provided an opportunity to assess the feasibility, content validity, and preliminary construct coherence of selected newly	Edits made throughout the manuscript.

	can support. Consider using more cautious wording such as “early validation,” “pilot validation,” or “initial validation of selected components.”	developed and adapted components within the broader survey instrument. **In response to the reviewer's suggestion, we have revised the manuscript throughout to consistently emphasise that the reported findings represent preliminary validation evidence from pilot testing of selected components** rather than psychometric validation of the complete 216-item survey tool. This distinction is now made explicit throughout the manuscript. Throughout the manuscript, we use revised wording to emphasise coherence, feasibility, and preliminary validation evidence rather than full psychometric validation of the survey tool.	
	**Reviewer 1: Comment 3:** Third, from a structural perspective, the manuscript at times conflates methods, results, and discussion. Certain sections appear to mix methodological description and interpretation without clearly presenting the underlying results, making it difficult to assess the evidence supporting the conclusions.	We do agree that the complexity of the survey development process required a clearer and more structured presentation; and have now restructured to ensure clearly defined sections to improve transparency and readability. The background and aims sections were revised to establish a clearer rationale for the survey tool and to align with the methodological objectives of the paper. The methodology section was restructured by assigning sub-sections describing the conceptual framework, overall survey development process, item generation procedures, content validation, pilot testing, and preliminary validation activities.	Throughout the manuscript
	**Reviewer 1: Comment 4:** Finally, the reporting of the entire tool development process, including item generation, validation, and testing, appears somewhat fragmented and difficult to follow. Given the complexity of these steps, the current presentation may be too dense or ambitious for a single paper, and could benefit from clearer structuring or even consideration of separating phases of the development process. Taken together, these issues limit the transparency and interpretability of the study. I recommend revising the manuscript to ensure (1) a clear and detailed description of all methodological steps, (2) full alignment between aims, methods, and results, (3) a clearer separation between		

	results and their interpretation, and (4) a more structured presentation of the tool development process.		
	Reviewer 2 - 7. Several sentences are long and difficult to follow. Careful language editing would improve readability.	Thank you for this suggestion. We have edited the language for clarity, readability, sentence structure, and flow. Long and complex sentences were revised and simplified where appropriate.	Throughout the manuscript
	Reviewer 2 - 8. Please use a consistent name for the instrument throughout the manuscript	Thank you for highlighting this. We have revised this and have now consistently used GEMMS survey tool throughout the manuscript.	Throughout the manuscript
	Reviewer 2 - 5. Key terms such as “gendered violence,” “precarity,” “governmentality-based precarity,” “structural harm,” and “sociocultural marginalisation” should be defined clearly and used consistently.	Thank you for highlighting this, and based on your suggestion, we have addressed it with clarity and consistency in the Conceptual framework section and then finally in the results section.	Throughout the manuscript
	Reviewer 2 - 6. Some sections of the background and discussion repeat similar points about structural vulnerability, syndemics, and intersectionality. The manuscript would benefit from some tightening.	Thank you for this observation. We have carefully reviewed the manuscript and revised the background and discussion sections to reduce repetition, and strengthen the overall flow of the argument.	Throughout the manuscript
**Methodology**	**Reviewer 1: Comment 1:** Across sections, there appears to be a lack of alignment between the study aims, methods, and reported results. First, the methods section does not provide sufficient detail to ensure reproducibility. Key processes, such as the development of the tool items and the content validation procedure, are insufficiently described, and no clear methodological framework is specified (e.g. Delphi method, Content Validity Index approach).	Thank you for this important observation. We have substantially revised the manuscript to improve alignment between the background, aims, methods, and results, and to strengthen methodological transparency and reproducibility. The methodology section has been extensively reorganised and expanded to improve methodological transparency, strengthen coherence across manuscript sections, and facilitate reproducibility of the survey development process.	Methodology section
	**Reviewer 2: Comment 2:** The exploratory factor analysis needs fuller reporting.<br>The factor analysis is one of the main empirical findings of the paper, but the current description is not detailed enough for readers to judge the robustness	Thank you for highlighting this. Given the length of the paper, we had provided limited information and agree that it limits assessment of robustness. We have now revised the methods and results section to include: the	See Factor Analysis under Methodology, and Construct Coherence under results

	of the results. Please provide more information on the number of items entered into each analysis, the effective sample size after missing data and skip patterns, extraction method, rotation method, criteria for deciding the number of factors, loading thresholds, and how cross-loadings were handled. It would also be useful to report whether factorability was assessed, for example using KMO and Bartlett's test. Given the relatively small pilot sample and the multi-country nature of the data, the limitations of the factor solution should be discussed more explicitly.	number of items entered in each analysis, effective sample size after accounting for missing data and skip patterns, data extraction and rotation method, criteria for selecting the number of factors and loading thresholds, factorability assessment using Kaiser-Meyer-Olkin measure, and procedures for handling cross-loadings. We clarify that we conducted EFA only on selected, adapted de novo multi-item scales lacking prior validation in the study context. EFA results tables are now updated and the limitations section expanded to clarify the scope and what these findings represent.	
	**Reviewer 2: Comment 3:** Please explain more clearly how cross-country comparability was maintained.<br>One of the most interesting features of the tool is the balance between standardisation and local adaptation. However, it is not always clear which items were identical across sites, which were adapted linguistically or culturally, and which were country-specific. This distinction is important because the tool is intended to support comparison across different migrant populations and settings. A clearer explanation, preferably with a summary table, would help readers understand how comparability was preserved despite necessary contextualisation.	Thank you for highlighting this. We have now revised the Methods section to clarify how we preserved comparability across sites. We differentiate core standardised items, linguistically or culturally adapted items, and locally required country specific response categories. A new summary table shows how different sections of the survey tool were handled. This added explanation clarifies that: i) comparability was preserved at the level of domains, constructs, item intent, recall period and response structures.<br>ii) contextualisation was used to improve comprehension, relevance and validity in specific settings; and iii) country-specific items were retained solely to support country-level interpretation and future within-country intersectional analysis, not for cross-country prevalence comparisons.	New sub-heading introduced before Factor Analysis under Methodology
	**Reviewer 2: Comment 4:** The rationale for treating the gendered violence module as an event inventory should be strengthened in the main text.	Thank you for this helpful feedback. We have strengthened the description of the GV module In the methods section, under	See Items for GV section, and content validation

	I agree with the authors' decision not to treat the gendered violence section as a reflective psychometric scale. The module appears to be designed to capture discrete events, perpetrators, settings, frequency, and help-seeking responses, rather than a single latent construct. This is methodologically appropriate. However, this point should be stated more clearly in the main manuscript.<br>The authors should also describe how the quality of this section was assessed, for example through content validity, acceptability, routing logic, ethical safety, and alignment with established gender-based violence assessment frameworks.	'Items for the gender-based violence section of the survey tool'. we now state explicitly the same.<br>We also detail the quality assessment steps: the GV section was informed by established GBV assessment frameworks; expert review consultations with GBV specialists for content validity, community-based content validation, piloting to assess item acceptability and supported by routing logic (and skip patterns) to reduce respondent burden. These additions clarify our rationale and the procedures used to ensure the module's validity and ethical implementation.	
**Methodology**	**Reviewer 2: Comment 5:** The **content validation process** should be described more systematically.<br>The participatory development process is a major strength of the study, but the reporting of content validation could be clearer. Please provide more detail on who participated in each stage, how many people were involved, how relevance and comprehensibility were assessed, and how the final decisions were made. In particular, some items appear to have relatively low item-level CVI values. It would be helpful to explain whether these items were revised, retained, removed, or further discussed, and what decision rules were used.	We thank the reviewer for this helpful observation and agree that the original manuscript did not provide sufficient detail regarding the content validation process. We have substantially expanded the Content validation section to describe the multi-stage participatory development and validation approach, the participants involved in each stage, the assessment procedures used, and the decision-making process for items revised. These additions enhance the transparency, reproducibility of the content validation process, and show how lower CVI values were handled through revision, review	See Content validation section
	**Reviewer 2: Comment 6: Feasibility and respondent burden** require more attention. The final tool contains 216 items and includes sensitive topics such as violence, mental health, trauma, documentation insecurity, and suicidal ideation. Please report, if available, the approximate duration of administration, completion rates, interviewer feedback, participant	Thank you for this important observation. We have expanded the 'Pilot testing' and 'Survey tool refinement' sections with further details on operational feasibility, respondent burden reduction strategies, and other quality assurance and safeguarding considerations (e.g. privacy procedures, interviewer training, safeguarding measures, distress	See Content validation and Pilot testing

	burden, and any difficulties observed during the pilot.	management protocols, referral pathways, and implementation experiences) across participating countries.<br>We clarify how pilot implementation informed refinements to routing logic and administration procedures. While formal metrics on survey duration, completion rates, and respondent burden were not recorded, the duration and related challenges were discussed in daily debriefings for refinement and the final tool amended accordingly.	
	**Reviewer 2: Comment 7: Ethical safeguards** should be described in greater detail. Because the survey includes highly sensitive questions, the manuscript should provide more detail on the ethical procedures used during data collection. In particular, please describe how privacy was ensured during interviews, how interviewers were trained, what distress protocols were in place, how disclosures of violence or self-harm risk were managed, and what referral pathways were available. These details are essential for assessing the practical and ethical robustness of the tool.	Thank you for highlighting this important issue. We have substantially expanded the description of the ethical and safeguarding procedures used during pilot implementation.	Pilot testing and Survey tool refinement section
	Reviewer 2 - 4. Please explain how missing data were handled in the pilot analyses, especially in the factor analysis.	We have added these details in the revised section on EFA.	Factor Analysis
	2. A table summarising the nine sections of the tool, the number of items in each section, the item sources, and the type of validation conducted would improve readability.	These details are in the supplementary material and figure 2.	Supplementary material and figure 2
**Results**	Reviewer 2 - 3. Please provide more detail on the pilot sample, including the number of participants by country and key demographic characteristics.	This information was provided in table 2.	Table 2 in Results
	**Reviewer 1: Comment 2:** Second, there are inconsistencies between the methods and results sections. For example, results referring to	We have now revised and reorganised to move the methodology component of the piloting under the methodology section. We	Content validation

	a pilot study are presented without a corresponding description in the methods. Similarly, the justification of the sample size is based on criteria for pilot studies assessing face and content validity, whereas the stated aim is to assess construct validity. This raises concerns, as construct validity typically requires different methodological approaches and larger sample sizes.	also revised the terminology within the content validation section to ensure consistency with the procedures undertaken. Specifically, clarified sample-size rationale..; Replaced imprecise wording (.e.g 'constructs' in relation to the I-CVI exercise with 'survey domains'	
