# Supplementary material for: Developing a survey tool to assess gendered violence and mental health burden in migrants living in precarity
Source: BMJ Open. 2026 Sep 30;16(9):e120912. doi: 10.1136/bmjopen-2026-120912 (PMC13630092; doi:10.1136/bmjopen-2026-120912)
Supplement: Supplementary file 2 [file bmjopen-16-9-s001.docx]

## SUPPLEMENTARY MATERIAL

### Supplement 1 Measurement, Sources and Decisions

#### Developing the Background and Migration Section

Given the substantial heterogeneity in migrants’ contexts across the four study sites, developing a standardized background section proved to be most challenging components of survey tool development.

For example, agreeing on a harmonised question on schooling or level of education proved to be a major challenge due to different framing of education across the four countries, each indicating different numbers of years spent in the school, different labels of education. Another example was that of relationship status, where options of single and never had a partner were added, to capture cases where questions of intimate partner violence would be applicable even if the respondent were single at the time of the survey but may have had a past relationship.

In the migration section, capturing country-specific contexts was critical because migration in these settings is neither linear nor uniform, and includes patterns like temporary, circular, seasonal, cross-border, inter-state and urban-rural, each associated with very distinct forms of precarity and vulnerability.

EFA was conducted for de novo migration-related adversity items. For migration, 13 items were retained that reflected sociocultural marginalization, protection risks and threats, access to essential services, identity expression and everyday discrimination.

#### Developing MH and GBV Sections

Several MH tools were reviewed to capture anxiety, depression, post-traumatic stress disorder, quality of life and social connectedness. However, many were not considered due to the need for clinical administration and scoring requirements, length of the questions, and overlap with other items included in existing scales. These included Rosenberg Self-Esteem Scale (36), SF-36 (37), Social Provision Scale (38), Kessler K10 (39), DASS(40), PSQ (41), and the International Trauma Questionnaire(42). For domains like quality of life, EQ-5D-5L and MANSA were reviewed but not included (EQ-5D, 1990; Priebe et al., 1999).

To ensure alignment with established GV assessment and monitoring practice, the team cross-checked content against the RHRC Consortium manual, emphasizing multi-sectoral response, standardized incident documentation, and safe referral(34).

For GV, since the section structure is not a latent scale but an event or experience module, EFA and reliability testing were not done. These tests assume that latent constructs are being measured and the questions are on a reflective scale. Whereas in the GEMMS survey tool, the GV section is a screening and event-mapping inventory, with questions like ‘has this happened’, ‘who did this’, ‘how frequently did it happen’, ‘did you seek support’ etc. None of these items are interchangeable indicators of a single trait but distinct events. Additionally, as elaborated earlier, the violence section is heavily branched with multiple skip logics, making a consistent ‘**n**’ impossible. The purpose of the GV section was to estimate prevalence and unpack patterns, not creating a new scale.

For MH, items other than validated instruments were included in EFA.

### Supplement 2 Country Case Details - India

This supplement presents India case study, illustrating the participatory content validation process undertaken during the GEMMS survey tool development and community consultations for the same. The tables document how the local advisory group (LAG) inputs informed the linguistic refining at the item-level, as well as validated the content, demonstrating how local epistemologies and lived experiences were integrated into the survey design.

#### Supplementary Table S1. Item-level Content Validity Index (I-CVI) results from Local Advisory Group Consultations in India

Based on members’ ranking on relevance, clarity and comprehension. The members were informed that relevance referred to whether the item reflected a core construct within its domain; clarity assessed if the question was easy to understand and respond to; and comprehension focused on whether respondents, particularly migrants with varying educational and linguistic backgrounds, would interpret the item as intended.

|  | Questions | I-CVI | CVI Category |
| --- | --- | --- | --- |
| 1 | Has the majority of your family also been living here for less than ten years? | 1 | Perfect I-CVI (1.00) |
| 2 | What is your current age in years? | 1 | Perfect I-CVI (1.00) |
| 3 | Years of schooling completed | 1 | Perfect I-CVI (1.00) |
| 4 | Do you have enough food at home to eat each day? | 1 | Perfect I-CVI (1.00) |
| 5 | What type of place/structure are you living in? | 0.71 | Low I-CVI (< 0.78) |
| 6 | Has your household been affected by cyclones or floods, or other natural disasters in the last 12 months, resulting in loss of access to services and water, damaged toilet or home, etc.? | 1 | Perfect I-CVI (1.00) |
| 7 | What kind of contract do you have for your employment? | 0.88 | Acceptable I-CVI (>/= 0.78) |
| 8 | Have you accessed/benefitted from any of these government or other entities benefit schemes/programs/services? | 1 | Perfect I-CVI (1.00) |
| 9 | What were the main reasons for your migration here? | 1 | Perfect I-CVI (1.00) |
| 10 | Who facilitated your migration? | 1 | Perfect I-CVI (1.00) |
| 11 | Are you continuously facing threats to your life here? | 1 | Perfect I-CVI (1.00) |
| 12 | Do you have access to quality healthcare facilities without discrimination? | 0.75 | Low I-CVI (< 0.78) |
| 13 | Do you use any drugs or substances other than those required for medical reasons? | 0.88 | Acceptable I-CVI (>/= 0.78) |
| 14 | (Violence - Males only): Do you live in fear of your safety/ Do you feel unsafe? | 0.88 | Acceptable I-CVI (>/= 0.78) |
| 15 | (Violence - Males only): Have you ever been slapped/ hit / pushed / beaten? | 0.88 | Acceptable I-CVI (>/= 0.78) |
| 16 | (Gender-Based Violence- single/never partnered females only): Have you ever experienced unwanted sexual acts, such as inappropriate touching or groping by someone? | 0.875 | Acceptable I-CVI (>/= 0.78) |
| 17 | (Gender-Based Violence-single/never partnered females only): Who did this? | 0.714286 | Low I-CVI (< 0.78) |
| 18 | (Gender-Based Violence - All Other Ever Partnered Females): How frequently have they physically forced you to have sexual intercourse when you did not want to in last 12 months? | 0.77 | Low I-CVI (< 0.78) |
| 19 | Feeling nervous, anxious, or on edge | 0.875 | Acceptable I-CVI (>/= 0.78) |
| 20 | Not being able to stop or control worrying | 0.77 | Low I-CVI (< 0.78) |
| 21 | Thoughts that you would be better off dead or hurting yourself in some way | 0.87 | Acceptable I-CVI (>/= 0.78) |
| 22 | Are you always under the threat of arbitrary arrest and detention? | 0.77 | Low I-CVI (< 0.78) |
| 23 | Are you always under the threat of torture and inhuman treatment? | 0.77 | Low I-CVI (< 0.78) |
| 24 | In the past month, have you had nightmares about the violent events you experienced during migration? | 0.77 | Low I-CVI (< 0.78) |

This table depicts selected examples of item-level wording revisions made after the structured content validation with Local Advisory Group members in India. These revisions focused on improving the overall comprehension of the survey tool, enhance its cultural resonance and acceptability among respondents from diverse literacy levels, while retaining conceptual intent of each item, and enhancing colloquial relevance.

#### Supplementary Table S2. Item-level language adaptations informed by Local Advisory Group Consultations in India

| Original Item / Term | Revised Term / Wording (as per LAG suggestion) | Rationale / Note |  |
| --- | --- | --- | --- |
| Aapke saath kaun rehta hai? | Yahan aap ke saath kaun-kaun rehta hai? | ‘Aapke saath’ sounded interrogative; revised for warmth and ease | |
| Sthalantran | Pravas | ‘Pravasi’ is more locally understood; less technical | |
| Sanstha/Samarthan/Sambandh | Dosti, pehchan, madad, aaju-baaju | Colloquial terms improve comprehension for low-literacy respondents | |
| Tanav/Chinta | Tension, fikar, pareshani | English/Hindi mix reflects real-life usage and acceptability | |
| Naukari ka contract | Kaam ka contract | ‘Kaam’ better reflects informal work; ‘naukari’ implies formal jobs | |
| Laingik hinsa | Galat kaam, ched chad, gandi baat | Alternative vocabulary increases acceptability for sensitive topics | |
| Mental Health (Maansik Swasthya) | Dimag katna, tension, khudko haani, bure khayal | Lived-experience-based expressions of distress are more relatable. | |

### Supplement 3 Full Survey Questionnaire

#### Eligibility Screening

001	Are you 18 yrs of age or above?

I002	Have you been living in this area / city/ town for less than ten years?

003	Do you live here with your family?

004	Has majority of your family also been living here for less than ten years

005	Did you move here for work, refuge or living/ better life?

#### Section I Background Characteristics (11)

Thank you for agreeing to be part of this survey. In this section, we will ask you questions about your background characteristics, economic situation, and employment conditions. Your responses will provide important context for our study and help us understand the diverse experiences of participants.

101	What is your gender?

102	What is your current age completed in years?

103	What is the highest level of schooling you have completed?

104	What religion do you follow/ practice?

105	Social Group (For India only)

106A	Language Spoken at home (For South Africa and Zimbabwe)

106B	Languages spoken at home (For India and Cambodia)T

107	Ethnicity

108	Mention the state/ province and country you were born in?.

109	What is your relationship status

110	How many children do you have?

111	How many of these are living with you

#### Section II Socioeconomic Status (6)

Thank you for your responses so far. I will now ask about some questions about your day to day living. Your responses will provide important context for our study and help us understand the diverse backgrounds of participants. I know that it is often difficult to talk about money as it is a very personal issue but I would like to understand your current situation

201	Have you borrowed money from others in last 12 months?

202	For what reason?

203	Do you have any savings currently?

204	What type of savings is it?

205	Thinking about last one year, did you have enough food at home to eat each day?

206	Have you ever had any food shortage in the house in last one year?

#### Section III Housing and Living Conditions (10)

Now I would like to ask you some questions related to conditions of your present space of living/house.

301	What type of house/ structure are you living in?

302	How many people (including yourself) are currently living with you in your residence? (When I ask about people living with you, I mean that in the place where you are living, there may be different people renting/living there who share the space/ kitchen/bathroom/yard & return to this when necessary).

303	Can you tell me the type of ownership of your place of living?

304	Main Material of the roof

305	Main Material of the exterior wall

306	Main material of the floor

307	Do you/ your household members own the following items that I will read out?

308	Do you/ your household members have a toilet facility to use?

309	What type of fuel does your household use for cooking?

310	Has your household been affected by cyclones or floods, or other natural disasters in the last 12 months, resulting in loss of access to services and water, damaged toilet or home, etc?

#### Section IV Employment and Work Conditions (30)

Thank you so much. I will now move to questions about your work. Please rest assured, your confidentiality will be protected throughout, and your responses will help us in understanding the circumstances under which migrants like yourself work.

401	Are you currently working i.e. earning money?

402	Were you working / earning money in last six months?

403	What is/was your primary job here?

404	In addition to this, are there any other jobs that you do here?

405	What best describes your nature of work?

406	Do you work from home?

407	In one month, approximately how much money do you make (in local currency)

408	What kind of contract do you have for your employment?

409	For how long have you been working like this / in this current position?

410	How frequently do you get paid?

411	Are you in a payroll system? (Payroll system means regular payment, employer deductions, tax deductions

412

What is the mode of payment of your salary/ income?

413	How do you sustain yourself here?

414	Do you have drinking water facilities at your workplace?

415	Do you have toilet facilities at your workplace?

Q416	Are you entitled for any of the following benefits through your work?

417	You get sufficient breaks during your working hours.

418	You are allowed to take time off to visit your native place when need arises.

419	You receive adequate support from your boss/supervisor.

420	If you lose your current work then how soon do you expect to find another job?

421	What types of dangers are involved in the work you are usually engaged in?

422	Are you able to complain about dangerous work or hazardous workplace conditions to any authority within your organization?

423	Are you provided with all safety measures at your workplace.

424	Does your work affect your physical health badly?

425	Have you ever suffered from any accident at the workplace leading to an impairment/ physical injury?

426	What kind of injury/ impairment?

427	Have you received compensation or care for it?

428	What kind of compensation/ care did you receive other than money?

429	Have you ever worked in the past?

430	Does your partner work?

#### Section V Access to State Benefits & Humanitarian assistance (3)

In this section, we will ask you about any benefits or schemes or programs through which you may have received support.

501	Are you aware of any of these government or other entities’ benefit schemes/ programs/ services?

502	Have you accessed/ benefitted from any of these government or any other entities’ benefit schemes/ programs/ services?

503	If you cannot access any of the schemes that you know of, what are the reasons?

#### Section VI Migration and Assimilation(29)

Now I would like to ask you a few questions about your migration history and your experience of living through your migration journey.

601	When did you first move here (this place of enumeration)?

602	When did you most recently move here? Enter number of months.

603	From which place/location/have you migrated? Mention village or town, city / district, state/ province, country)

604	How frequently do you cross any international border (e.g. SA-Zim or SA- Mozambique)?

605	Do you now live permanently here in this city?

606	In a year how many months do you normally (expect to) stay here?

607	How many times have you moved/ migrated within this city in last one year?

608	How many times have you moved / migrated outside of this city in last one year?

609	What were the main reasons for your migration here?

610	Who had facilitated your move / migration to this place?

611	Who did you travel/move with?

612	How did you travel to this place?

613	What kind of identification document/ travel document did you have while travelling here?

614	Who had your identification/ travel documents while you were migrating here?

615	How did you pay for your journey to your present location?

616	Who did you make this payment to?

617	Do you understand the language(s) of the locations you are currently living in?

618	You always living in the fear of arbitrary arrest and detention.

619	You are always living in the fear oftorture and inhuman treatment.

620	You are always under a fear of being expelled or deported from here.

621	You face discrimination while accessing quality health care facilities.

622	You are unable to enjoy your cultural practices.

623	You are unable to freely practise your religion..

624	You are unable to express yourself in your language.

625	You are treated with less respect than other people are.

626	You receive poorer service than other people at restaurants or stores.

627	You are called names or insulted.

628	You are treated with suspicion in public spaces

629	You were you lied to, tricked/ deceived, manipulated or given false promises in order to get you to travel/move?

#### Section VII General Health and Health Access (18)

In this section, I will ask you a series of questions related to your general health. Your responses will help us gain valuable insights into health behaviours. Your confidentiality is assured so please answer as accurately as possible.

701	In general, how would you describe your overall health?

702	In the past month, have you been in any pain and/or discomfort?

703	Do you suffer from any disability?

704	Do you suffer from any of these chronic health conditions / diseases?

705	Are you taking any medical treatment for any of the conditions you just mentioned?

706	From where are you/ would you normally/ usually access the treatment/ care you need?

707	Are you able to access any public sector(government) health care facility when needed?

708	Why can you not access this health care facility?

709	Do you drink alcohol

710	How often do you drink alcohol?

711	Do you use any drugs or substances other than those required for medical reasons?

712	How often do you use drugs or substances other than those required for medical reasons?

713	What do you use/take?

714	Does your partner drink alcohol?

715	How often does your partner drink alcohol?

716	Does your partner use any drugs or substances other than those required for medical reasons?

717	How often does your partner use drugs or substances other than those required for medical reasons?

718	What do they use/take?

#### Section VIII Violence

Thank you for continuing with this survey. In this section, we will ask about any difficulties you've experienced due to being subjected to different forms of violence.

Your responses are crucial for understanding these impacts. Please know your confidentiality is protected, and you can stop at any time if you feel uncomfortable & also indicate to move on to the next question

801A	Do you live in fear of your safety/ Do you feel unsafe?

802A	What are the primary reasons you do not feel safe in your current environment?

803A	Have you ever been physically hit?

804A	Who has hit you?

805A	Have you experienced any of the following forms of economic/ financial situations/ violence?

806A	How frequently has this occurred in last 12 months?

807A	Who did this?

808A	Who did you report to?

809A	Was any action taken on your report?

810AI	Have you ever been insulted in public, repeatedly humiliated, shouted at, threatened and/ or intimidated?

811A	How frequently has this occurred in last 12 months?

812A	Who did this?

813A	Have you ever experienced sexual assault/ abuse?

814A	Approximately, how frequently has this happened in last 12 months?

815A	Who did this?

816A	Who did you seek support from?

817A	Did you feel helped?

818A	Have you ever been pressured or forced to engage in violent or risky behaviors to "prove" your masculinity?

819A	What kind of behavior/ activity was this?

I will start with asking about some questions about your experiences with your partner, to which you can simply respond in yes or no.

803C	Do/did they try to keep you from seeing your friends

804C	Do/did they try to restrict contact with your family of birth

805C	Do/did they insist on knowing where you are at all times?

806C	Do/did they ignore you and treat you indifferently

807C	Do/did they get angry if you speak to another man

808C	Are/ were they often suspicious that you may be unfaithful

809C	Do/did they expect you to ask their permission before seeking healthcare for yourself.

Thank you for sharing that. I will now proceed to ask about more upsetting incidents that you may have experienced with your partner.

810C	Have they ever insulted you or made you feel bad about yourself?

811C	How frequently have they insulted you or made you feel bad about yourself in last 12 months?

812C	Have they belittled you or humiliated you in front of other people?

813C	How frequently have they belittled you or humiliated you in front of other people in last 12 months?

814C	Have they done things to scare or intimidate you on purpose?

815C	How frequently have they done things to scare or intimidate you on purpose in last 12 months?

816C	Have they threatened to hurt you or someone you care about?

817C	How frequently have they threatened to hurt you or someone you care about in last 12 months?

818C	Have they slapped you or thrown something at you that could hurt you?

819C	How frequently have they slapped you or thrown something at you that could hurt you in last 12 months?

820C	Have they pushed you or shoved you?

821C	How frequently have they pushed you or shoved you in last 12 months?

822C	Have they hit you with their fist or something?

823C	How frequently have they hit you with their fist or something in last 12 months?

824C	Have they kicked you, dragged you or beaten you?

825C	How frequently have they kicked you, dragged you or beaten you in last 12 months?

826C	Have they choked or burnt you on purpose?

827C	How frequently have they choked or burnt you on purpose in last 12 months?

828C	Have they threatened to use a gun, rod, knife or another similar etc.?

829C	How frequently have they done this in last 12 months?

830C	Have they physically forced you to have sexual intercourse when you did not want to?

831C	How frequently have they physically forced you to have sexual intercourse when you did not want to in last 12 months?

832C	Have you had sexual intercourse that you clearly didn’t want but were afraid of what they might do if you refused?

833C	How frequently have you had sexual intercourse that you clearly didn’t want but were afraid of what they might do in last 12 months?

834C	Have they forced you to do something sexual that you found degrading or humiliating?

835C	How frequently have they forced you to do something sexual that you found degrading or humiliating in last 12 months?

836C	For any of these above-mentioned incidents/ experiences, who did you seek help/support from?

837C	Did you feel helped?

838C	How frequently have any of these incidents resulted in an injury?

839C	What type of injuries resulted from any of these incidents?

840C	For any of these injuries, who did you seek help/support from?

841C	Did you feel helped?

Thanks for sharing all your experiences with me. I truly appreciate this. I will now proceed to asking about similar incidents that you may have experienced, but with people other than your partner.

842C	Have you experienced physical violence outside home?

843C	Who did this?

859C	Have you ever been pregnant?

860C	How many times have you been pregnant

861C	Did you ever have a pregnancy that was miscarried, ended in still birth or an abortion?

862C	Did you ever have any unwanted pregnancy?

863C	Have you ever been mis/ illtreated during childbirth?

864C	What form did that take?

865C	Where did this happen?

866C	Who did this?

867C	Who did you seek help/ support from?

868C	Did you feel helped?

869C	Has your husband/partner ever refused to use a condom?

870C	Has your husband or partner ever stopped you from taking any form of contraception?

#### Section IX Mental Health (39)

I appreciate you continuing to participate in this survey so far. I now have a few questions/ statements about your mental health to better understand any challenges you may be facing. Your responses are completely confidential and will not be shared with anyone. If you prefer not to answer a question, please let me know, and we will move on to the next one. I will be now asking you about some feelings you may have had in the last one month. You may say not at all if you have never had such feelings. If you have experienced this at least 4-12 days during last one month, you can select several days. If you have experienced this on most days like at least 14-24 days in last one month, you can select more than half the days, and if you have experienced it everyday, please select everyday. I hope this is clear.

How frequently are you:

901	Feeling nervous, anxious, or on edge

902	Not being able to stop or control worrying

903	Worrying too much about different things

904	Trouble relaxing

905	Being so restless that it’s hard to sit still

906	Becoming easily annoyed or irritable

907	Feeling afraid as if something awful might happen

908	Little interest or pleasure in doing things

909	Feeling down, depressed, or hopeless

910	Trouble falling or staying asleep, or sleeping too much

911	Feeling tired or having little energy

912	Poor appetite or overeating

913	Feeling bad about yourself — or that you are a failure or have let yourself or your family down

914	Trouble concentrating on things, such as reading the newspaper or watching television

915	Moving or speaking so slowly that other people could have noticed, or being so fidgety or restless that you've been moving around a lot more than usual

916	Thoughts that you would be better off dead or hurting yourself in some way

917	Have you ever experienced this kind of event?

A physical assault or abuse..1

A sexual assault or abuse ..2

Torture or other forms of violence..3

Separation from family..4

Destruction of home/ livelihoods..5

Xenophobia and discrimination..6

Detention and deportation trauma..7

Exposure to community /armed conflict/ bombings/ shootings/war..8

Having a loved one die through homicide or suicide..9

Seeing someone be killed or seriously injured ..10

Life-threatening conditions during migration/movement..11

Exploitation by smugglers/ traffickers/ employers

A serious accident or fire ..12

An earthquake or flood ..13

Any other untoward incident during your journey to the destination..14

None of these..15

918	In the past month, have you had nightmares about the event(s) or thought about the event(s) when you did not want to?

919	In the past month, have you tried hard not to think about the event(s) or went out of your way to avoid situations that reminded you of the event(s)?

920	In the past month, have you felt guilty or unable to stop blaming yourself or others for the event(s) or any problems the event(s) may have caused?

921	In the past month, have you been constantly on guard, watchful, or easily startled?

922	In the past month, have you felt numb or detached from people, activities, or your surroundings?

923	You feel close to people in this community.

I will now ask you some questions about your adjustment in this place/ country/ state that you have migrated to?

924	You feel a sense of belonging with others in this place.

925	You don’t feel involved with people around you. (reverse-scored)

926	You feel like you fit in when you are with other people in this place.

927	You are able to relate to others around you.

928	You feel isolated from people in your host place.

929	You don’t feel connected to anyone here.

930	You have difficulty understanding the customs of this place where you have migrated to.

931	You feel like you do not belong in this place.

932	You have been treated unfairly because you are a foreigner/ not from this place

933	You feel that others look down on you because of your background.

934	It is hard for you to make friends with people from the local community/ people who are originally from this place

935	You often feel like an outsider in social settings.

936	You miss your family and friends from your home country/ place where you came from.

937	You did not expect that it was so difficult to make money here

938	Most things did not go as planned

939	Nothing here is the same as you thought of before you came

#### Section X Future Studies (2)

Thank you so much for your time and patience. We are the end of the survey now.

1001	Would you be interested in opting for further set of few questions in 6-12 months from now?

1002	Message: Please note down the respondent’s name and contact information in your physical notebook. Once you have noted the details, click next.

#### Section XI For Enumerator/ Surveyor only (1)

1101	Based on the interview, and your assessment of the lived experiences of this respondent, their GBV experience and their mental health responses, do you think this respondent would be a potential participant for qualitative research methods.

#### Concluding Script

Thank you for being part of the survey and we really appreciate the time you have taken to be with us to answer this. Feel free to get in touch if you still have any questions. Thank you once again.

### Supplement 4 Skip and Routing Summary

This supplement summarises the routing rules and skip logic embedded in the digitised GEMMS survey questionnaire. The survey was administered using an interviewer-led, tablet-based platform with automated skips to minimise respondent burden and ensure ethical administration of sensitive modules.

##### 1. Consent and Eligibility Routing

If informed consent is not provided, the interview is terminated.

Eligibility screening is country-specific:

South Africa, Zimbabwe, Cambodia: eligibility requires residence in the current location for <10 years.

India: eligibility requires residence in the current location for <20 years.

Respondents are excluded and the interview terminated if:

- Age <18 years.
- Migration duration exceeds the country-specific threshold.
- Migration was not undertaken for work, refuge, or living/better life.
- Household composition questions are skipped if the respondent does not live with family.

##### 2. Country-Specific Routing

The following items are displayed only for relevant countries:

India only:

- Social group/caste classification.
- Open-ended ethnicity item.

South Africa and Zimbabwe only:

- Language spoken at home (multi-select).
- Cross-border migration frequency questions.

India and Cambodia only:

- Language spoken at home (open-ended).

All other country-specific items are automatically hidden for non-eligible countries.

##### 3. Employment and Work Conditions Routing

- If the respondent is not currently working:
  - Current employment questions are skipped.
  - Past work history questions are displayed.
- Work-related facilities (toilet, drinking water, payroll, benefits) are skipped if:
- The respondent is self-employed, unpaid family worker, or informal worker, where not applicable.
- Workplace injury follow-up questions are shown only if the respondent reports an injury.
- Compensation and care questions are shown only if an injury was reported and the respondent was eligible for compensation.

##### 4. Partner-Related Routing

Questions involving partner behaviours (alcohol use, substance use, work status) are displayed only if the respondent reports being:

- In a relationship,
- Cohabiting,
- Married.

These items are skipped for respondents who are single and never partnered.

5. Migration History and Assimilation Routing

- Cross-border movement questions are displayed only for respondents in South Africa or Zimbabwe.
- Questions on months of stay, frequency of movement, and mobility history are displayed only if the respondent does not report permanent residence at the destination.
- Documentation-related follow-up questions are skipped if the respondent reports having no documents or does not know their document status.

6. Violence Module Routing

Violence modules are mutually exclusive and triggered based on gender and relationship status:

- Section VIIIA (Violence – males only) Displayed if gender = male.
- Section VIIIB (Violence – never-partnered females) Displayed if gender = female and relationship status = never partnered.
- Section VIIIC (Gender-based violence – ever-partnered females) Displayed if gender = female and relationship status indicates current or past partnership.

Within all violence modules:

- Event-specific follow-up questions (frequency, perpetrator, reporting, action taken) are displayed only if the corresponding violence experience is reported.
- Help-seeking and outcome questions are skipped if no reporting or support-seeking occurred.
- Reproductive and obstetric violence questions are displayed only for ever-pregnant respondents.

##### 7. Health and Mental Health Routing

- Substance use frequency questions are displayed only if substance use is reported.
- Partner substance use questions are displayed only if the respondent has a partner.
- Trauma symptom questions (PC-PTSD items) are displayed only if at least one traumatic event is endorsed.
- Mental health symptom items (PHQ-9, GAD-7) are administered to all respondents without skips, except for refusal or withdrawal.

##### 8. Access to Benefits Routing

- Questions on utilisation of state or humanitarian benefits are displayed only if awareness of at least one scheme is reported.
- Reasons for non-access are displayed only if the respondent reports awareness but no utilisation.

##### 9. Follow-up and Enumerator Assessment Routing

- Consent for future contact is requested only at the end of the survey.
- Enumerator assessment of suitability for qualitative follow-up is not visible to respondents and is completed post-interview.

##### 10. Ethical Safeguards in Routing

- Respondents may refuse any question without penalty.
- Sensitive modules (violence, mental health) include the option to skip or terminate at any time.
- All skip logic was automated in the digital platform to prevent interviewer error and reduce re-traumatisation risk.
