# Supplementary material for: Developing a survey tool to assess gendered violence and mental health burden in migrants living in precarity
Source: BMJ Open. 2026 Sep 30;16(9):e120912. doi: 10.1136/bmjopen-2026-120912 (PMC13630092; doi:10.1136/bmjopen-2026-120912)
Supplement: Supplementary file 3 [file bmjopen-16-9-s002.docx]

# TWO CHECKLISTS APPLIED

## A. COSMIN for content validation, pilot testing, and selective psychometric assessment of some item sets

**Relevant Items**

### A1. General Reporting recommendations relevant for all studies on measurement properties

| **Item Number** | **Item Name** | **Item Description** |  |
| --- | --- | --- | --- |
| **Report section: Title** | |  |  |
| T1 | Patient Reported Outcome Measure (PROM) | The name of the PROM instrument(s) (and version if relevant) being studied | 1-3 |
| T2 | Measurement Property (MP) | What MPs are being studied or more generally, that MPs are being studied (if there are many properties being investigated, for example) | 10-15 |
| T3 | Study sample | General description of relevant study sample characteristics (e.g., condition of interest, language) and also any intervention or exposure (e.g., treatments) if applicable. | 17 |
| **Report section: Introduction** | |  |  |
| I1 | Name and describe the PROM of interest | Specify the name, type, language, and version of the PROM being investigated and how it was developed. Describe the construct the PROM aims to measure and its subscales; describe the structure of the PROM (e.g., the number of factors, the number of items, scoring algorithm); describe relevant instructions (like time period), and number or type of response categories. State whether the PROM is based on a reflective or formative model. Note: This information may also appear in the methods section in greater detail. | 10-16 |
| I2 | Target population | Describe the specific target population that the PROM was designed for. The authors need to provide the appropriate and necessary characteristics of this population. | 17 |
| I3 | Citation for the original development of the PROM | The citation for the original development paper(s) should be provided and other highly relevant citations related to the quality of the specific PROM under investigation. | 27 |
| I4 | State of Knowledge & Rationale | A description of the current scientific knowledge (what is known) regarding the MPs of? the PROM under investigation. The authors should provide a literature review or refer to a recent review of all existing evidence of the specific version (e.g., language, short form) of the PROM and explain why the new study is necessary and important. The rational for the current proposed study should be given. | 6-8 |
| I5 | Definitions | Specialized terms should be defined or explained. | 6 |
| I6 | Objectives and Hypotheses | State the specific objective(s) of the research and hypotheses related to the specific PROM under investigation. | 8 |
| **Discussion** | | |  |
| D1 | MP evidence | Per measurement property the authors should compare the result to the criteria for good measurement properties (e.g., COSMIN criteria)[27], and determine if the specific MP is sufficient or not. Note: This information may also appear in the results section in greater detail in a table for example. | 23-24 |
| D2 | Practical relevance | The authors need to discuss the practical relevance of the findings. | 24 |
| D3 | Strengths and limitations | Strengths and limitations of the study should be discussed. For example, discuss if there were any significant potential biases in the study that could have impacted the results. | 4 |
| D4 | Generalizability | Generalizability issues related to the PROM results should be discussed. For example, discuss if the results could be generalized to other populations given the sample studied. | 25-26 |
| D5 | Instrument changes | Discuss the need for modifications to the existing PROM or new PROM development. If you conclude that one of the measurement properties is insufficient, you could suggest some modification, or if it is really poor, you could suggest stopping use of the PROM (in the specific population or in general). | 11 |
| D6 | Future Research | Report specifically the type of research needed to answer new questions arising out of these findings for the particular MP and PROM investigated. | 26 |
| Report Section: Conclusion | | |  |
| C1 | Conclusions | State the overall conclusions for each MP and of the use PROM investigated. | 26 |
| Report Section: Conflict of Interest | | |  |
| O1 | Conflict of Interest | State any relevant conflict of interest related to the PROM under investigation (e.g., an author being the PROM developer, funding body etc). | 4 |

### A2. Specific Reporting recommendations for studies on Content Validity

| **Item Number** | **Item Name** | **Item Description** |  |
| --- | --- | --- | --- |
| CV1 | Relevance | Report if and how patients and/or professionals were asked whether each item is relevant for their experience with the condition | 12 |
| CV2 | Comprehensiveness | Report if and how patients and/or professionals were asked whether all key concepts are included | 15-17 |
| CV3 | Comprehensibility | Report if and how the comprehensibility of the PROM instructions, items, response options, and recall period was assessed | 15-17 |
| CV4 | Relevance results | Report if all items were considered relevant for the construct, population, and context of use of interest by patients and/or professionals | 15-17 |

### A3. Specific Reporting recommendations for studies on Structural Validity

| **Item Number** | **Item Name** | **Item Description** |  |
| --- | --- | --- | --- |
| SV1 | Factor Analyses: Classical Test Theory (CTT) PROMs | Report details of the methods and results for any exploratory or confirmatory factor analyses. State the rational for any explorative factor analyses (e.g., no clear a priori hypotheses). For CFA, describe and justify the factor structure of tested models. Methods and results for checking of the assumptions should be described, the method of estimation, goodness-of-fit statistics and cut-off points for good model fit, including factor loadings of best-fitting model. | 17-22 |

### A4. Specific Reporting recommendations for studies on Internal Consistency

| **Item Number** | **Item Name** | **Item Description** |  |
| --- | --- | --- | --- |
| IC1 | Unit of measurement | Report internal consistency methods and results for each unidimensional scale or subscale. Report all evidence or assumptions associated with unidimensionality. | 17-22 |
| IC2 | Continuous scores | Report Cronbach’s alpha or omega statistics. Report other statistics calculated for internal consistency of continuous scores. | 20-22 |

## B. CROSS for Survey design - Checklist for Reporting Of Survey Studies (CROSS)

| **Section/topic** | **Item** | **Item description** | **Reported on page #** |
| --- | --- | --- | --- |
| **Title and abstract** | | |  |
| Title and abstract | 1a | State the word “survey” along with a commonly used term in title or abstract to introduce the study’s design. | 1 |
|  | 1b | Provide an informative summary in the abstract, covering background, objectives, methods, findings/results, interpretation/discussion, and conclusions. | 3 |
| **Introduction** | | |  |
| Background | 2 | Provide a background about the rationale of study, what has been previously done, and why this survey is needed. | 6-8 |
| Purpose/aim | 3 | Identify specific purposes, aims, goals, or objectives of the study. | 8 |
| **Methods** | | |  |
| Study design | 4 | Specify the study design in the methods section with a commonly used term (e.g., cross-sectional or longitudinal). | 6 |
|  | 5a | Describe the questionnaire (e.g., number of sections, number of questions, number and names of instruments used). | 10-15 |
| Data collection methods | 5b | Describe all questionnaire instruments that were used in the survey to measure particular concepts. Report target population, reported validity and reliability information, scoring/classification procedure, and reference links (if any). | 10-15, 17-21, Supplement |
|  | 5c | Provide information on pretesting of the questionnaire, if performed (in the article or in an online supplement). Report the method of pretesting, number of times questionnaire was pre-tested, number and demographics of participants used for pretesting, and the level of similarity of demographics between pre-testing participants and sample population. | 17-21 |
|  | 5d | Questionnaire if possible, should be fully provided (in the article, or as appendices or as an online supplement). | Supplement |
| Sample characteristics | 6a | Describe the study population (i.e., background, locations, eligibility criteria for participant inclusion in survey, exclusion criteria). | 18 |
|  | 6b | Describe the sampling techniques used (e.g., single stage or multistage sampling, simple random sampling, stratified sampling, cluster sampling, convenience sampling). Specify the locations of sample participants whenever clustered sampling was applied. | 17-18 |
|  | 6c | Provide information on sample size, along with details of sample size calculation. | 17 |
|  | 6d | Describe how representative the sample is of the study population (or target population if possible), particularly for population-based surveys. | 17 |
| Survey  administration | 7a | Provide information on modes of questionnaire administration, including the type and number of contacts, the location where the survey was conducted (e.g., outpatient room or by use of online tools, such as SurveyMonkey). | 17 |
|  | 7b | Provide information of survey’s time frame, such as periods of recruitment, exposure, and follow-up days. | 18 |
|  | 7c | Provide information on the entry process:  –>For non-web-based surveys, provide approaches to minimize human error in data entry.  –>For web-based surveys, provide approaches to prevent “multiple participation” of participants. | 18 |
| Study preparation | 8 | Describe any preparation process before conducting the survey (e.g., interviewers’ training process, advertising the survey). | 17 |
| Ethical considerations | 9a | Provide information on ethical approval for the survey if obtained, including informed consent, institutional review board [IRB] approval, Helsinki declaration, and good clinical practice [GCP] declaration (as appropriate). | 17 |
|  | 9b | Provide information about survey anonymity and confidentiality and describe what mechanisms were used to protect unauthorized access. | 17 |
| Statistical  analysis | 10a | Describe statistical methods and analytical approach. Report the statistical software that was used for data analysis. | 15-23 |
|  | 10b | Report any modification of variables used in the analysis, along with reference (if available). | 14 |
|  | 10c | Report details about how missing data was handled. Include rate of missing items, missing data mechanism (i.e., missing completely at random [MCAR], missing at random [MAR] or missing not at random [MNAR]) and methods used to deal with missing data (e.g., multiple imputation). | NA |
|  | 10d | State how non-response error was addressed. | NA |
|  | 10e | For longitudinal surveys, state how loss to follow-up was addressed. | NA |
|  | 10f | Indicate whether any methods such as weighting of items or propensity scores have been used to adjust for non-representativeness of the sample. | NA |
|  | 10g | Describe any sensitivity analysis conducted. | NA |
| **Results** | | |  |
| Respondent characteristics | 11a | Report numbers of individuals at each stage of the study. Consider using a flow diagram, if possible. | NA |
|  | 11b | Provide reasons for non-participation at each stage, if possible. | NA |
|  | 11c | Report response rate, present the definition of response rate or the formula used to calculate response rate. | NA |
|  | 11d | Provide information to define how unique visitors are determined. Report number of unique visitors along with relevant proportions (e.g., view proportion, participation proportion, completion proportion). | NA |
| Descriptive  results | 12 | Provide characteristics of study participants, as well as information on potential confounders and assessed outcomes. | 18 |
| Main findings | 13a | Give unadjusted estimates and, if applicable, confounder-adjusted estimates along with 95% confidence intervals and p-values. | NA |
|  | 13b | For multivariable analysis, provide information on the model building process, model fit statistics, and model assumptions (as appropriate). | NA |
|  | 13c | Provide details about any sensitivity analysis performed. If there are considerable amount of missing data, report sensitivity analyses comparing the results of complete cases with that of the imputed dataset (if possible). | NA |
| **Discussion** | | |  |
| Limitations | 14 | Discuss the limitations of the study, considering sources of potential biases and imprecisions, such as non-representativeness of sample, study design, important uncontrolled confounders. | 23-25 |
| Interpretations | 15 | Give a cautious overall interpretation of results, based on potential biases and imprecisions and suggest areas for future research. | 25 |
| Generalizability | 16 | Discuss the external validity of the results. | 15 |
| **Other sections** | | |  |
| Role of funding source | 17 | State whether any funding organization has had any roles in the survey’s design, implementation, and analysis. | 4-5 |
| Conflict of interest | 18 | Declare any potential conflict of interest. | 4 |
| Acknowledgements | 19 | Provide names of organizations/persons that are acknowledged along with their contribution to the research. | 1-2 |
